# Supplementary material for: Assessing Genetic Algorithm-Based Docking Protocols for Prediction of Heparin Oligosaccharide Binding Geometries onto Proteins
Source: Biomolecules. 2023 Nov 9;13(11):1633. doi: 10.3390/biom13111633 (PMC10669598; doi:10.3390/biom13111633)
Supplement: Supplementary file 1 [file biomolecules-13-01633-s001.zip › biomolecules-2687368-supplementary.pdf]

#	Contents	Pg.
**Figure S1**	Four Major Classes of GAGs	S2
**Figure S2**	Major IdoA Puckers	S3
**Table S1**	Combinatorics of Proteins, Nucleic Acids, and Hp/HS	S4
**Table S2**	PDB Survey of Φ,Ψ for Hp/HS Structures Organized by Acid→Amine Linkage Type	S5-S11
**Table S3**	PDB Survey of Φ,Ψ for Hp/HS Structures Organized by Amine→Acid Linkage Type	S12-S17
**Figure S3**	Comparative structures of two heparin oligosaccharides bound to thrombin.	S18
**Table S4**	Metadata for all Structures in this Study	S19
**Figure S4**	Visuals for Rigid Dockings	S20-S21
**Figure S5**	RMSD for Rigid Dockings at 300 GA Runs	S22
**Figure S6**	RMSD for Flexible Dockings at 300 GA Runs	S23
**Figure S7**	Example of GOLD torsional histogram for a semi-rigid docking	S24
**Figure S8**	Φ,Ψ from Semi-Rigid Dockings for two di- and one trisaccharides	S25
**Figure S9**	Φ,Ψ from Semi-Rigid Dockings for three tetrasaccharides	S26
**Figure S10**	Φ,Ψ from Semi-Rigid Dockings for four pentasaccharrides	S27
**Figure S11**	Φ,Ψ from Semi-Rigid Dockings for four hexasaccharides	S28
**Figure S12**	Φ,Ψ from Semi-Rigid Dockings for two octa- and one hexasaccharide	S29
**Figure S13**	Φ,Ψ from Flexible Dockings for two di- and one trisaccharides	S30
**Figure S14**	Φ,Ψ from Flexible Dockings for three tetrasaccharides	S31
**Figure S15**	Φ,Ψ from Flexible Dockings for four pentasaccharrides	S32
**Figure S16**	Φ,Ψ from Flexible Dockings for four hexasaccharides	S33
**Figure S17**	Φ,Ψ from Flexible Dockings for two octa- and one hexasaccharide	S34
**Figure S18**	RMSD for Rigid Dockings at 300 GA Runs	S35
**Figure S19**	Comparison of changes in torsions from the native following docking	S36
**Figure S20**	Docked Poses from the 3 Protocols for 3 di-, 1 tri- and 2 tetrasaccharides	S36
**Figure S21**	Docked Poses from the 3 Protocols for 1 tetra-, 4 penta- and 1 hexasaccharides	S37
**Figure S22**	Docked Poses from the 3 Protocols for 3 hexa-, 2 octa- and 1 decasaccharides	S38

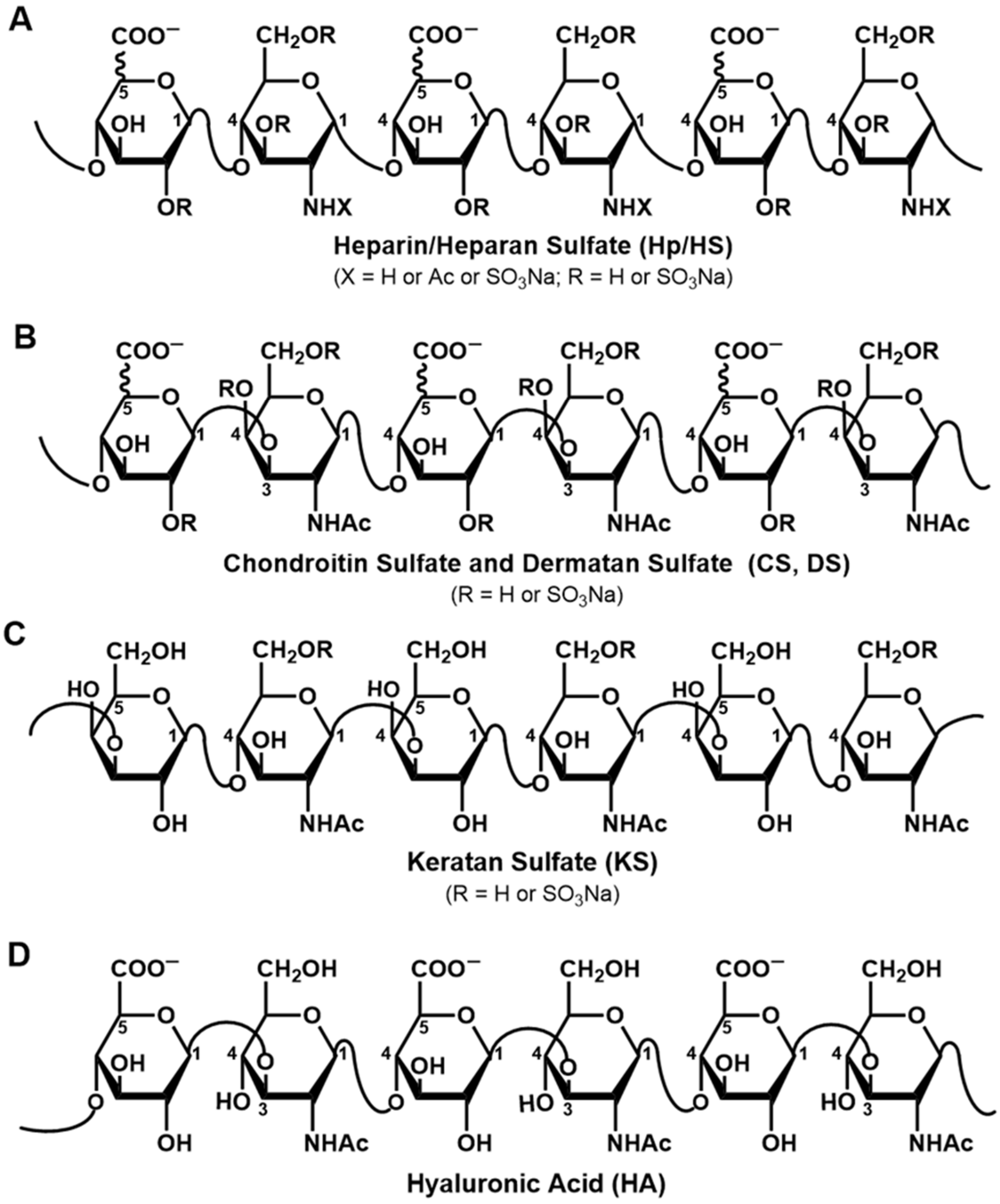

Figure S1. Four major classes of GAGs shown as Haworth projections. Red and blue indicates the two alternate linkages in each GAG. The curly bond between C5 and C6 in Hp/HS indicates the presence of either IdoA or GlcA in the disaccharide. In Hp/Hs, R_1 = H or SO_3Na and R_2 = Ac or SO_3Na. In all other GAGs, R = H or SO_3Na.

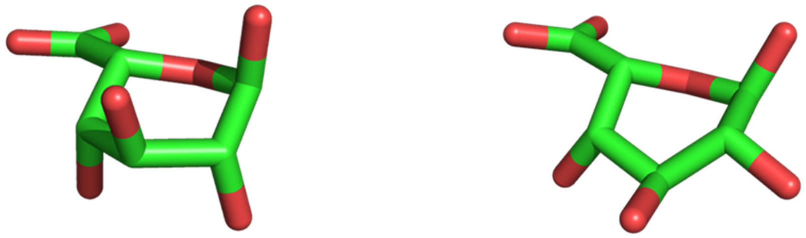

IdoA($^1C_4$) **IdoA(^2S_O)**

Figure S2. Two of the many possible conformations of iduronic acid (IdoA). Shown are $^1C_4$ and ^2S_O puckers, which typically are most populated in solution.

Table S1. Theoretical number of possible sequences/topologies for a hexameric repeating building block of biopolymers generated from nucleotides, amino acids and disaccharides. Note: The number of common building blocks increase when two most preferred puckers $^1C_4$ and ^2S_O are factored in.

Biopolymer	Number of Building Blocks	Number of Building Block Repeats	Combinatorial
Nucleic Acids	4	6	4^6 = 4,096
Peptides	20	6	20^6 = 64,000,000
Hp/HS	48#	6	48^6 = 12,230,590,464
Hp/HS	72 including IdoA in $^1C_4$ & ^2S_O forms	6	72^6 = 139,314,069,504

Theoretical number of disaccharide building blocks possible for Hp/HS biopolymers.

Table S2. Phi and psi corresponding to different glycosidic linkages of Hp/HS complexes with diverse proteins obtained from the protein data bank (PDB). Shown below are Φ and Ψ values for acid – amine linkages (UA→GlcN).

PDB	Length	Sequence	Linkage Type	Linkage Position	Φ	Ψ	Xray/NMR
3B9F	2	IdoA2S(2SO)-GlcNS	IdoA-GlcN	1	-67.8	-112.1	Xray
1U4L	2	ΔUA2S-GlcNS6S	ΔUA-GlcN	1	-96.1	64.1	Xray
1U4M	2	ΔUA2S-GlcNS	ΔUA-GlcN	1	-97.5	66	Xray
3OGX	2	ΔUA2S-GlcNS6S	ΔUA-GlcN	1	-112.4	-125.4	Xray
5DNF	3	GlcNS6S-IdoA2S(1C4)-GlcNS6S	IdoA-GlcN	1	-38.9	-85.3	Xray
3E7J	4	ΔUA-GlcNAc-GlcA-GlcNAc	GlcA-GlcN	1	-89.9	-112.8	Xray
1BFB	4	ΔUA2S-GlcNS6S-IdoA2S(1C4)-GlcNS6S	IdoA-GlcN	1	-62	-97.8	Xray
2HYU	4	ΔUA2S-GlcNS6S-IdoA2S(1C4)-GlcNS6S	IdoA-GlcN	1	-110.2	61.4	Xray
2VRA	4	IdoA2S(2SO)-GlcNS6S-IdoA2S(1C4)-GlcNS6S	IdoA-GlcN	1	-90.8	-98.9	Xray
3QMK	4	IdoA2S(1C4)-GlcNS6S-IdoA2S(1C4)-GlcNS6S	IdoA-GlcN	1	-76.5	-103.7	Xray
5E9C	4	ΔUA2S-GlcNS6S-IdoA-GlcNS6S	IdoA-GlcN	1	-24.7	-118.1	Xray
1T8U	4	ΔUA2S-GlcNS6S-IdoA2S(2SO)-GlcNS6S	IdoA-GlcN	1	-77.2	-133.5	Xray
2VRA	4	IdoA2S(2SO)-GlcNS6S-IdoA2S(1C4)-GlcNS6S	IdoA-GlcN	3	-81.6	-145.2	Xray
3QMK	4	IdoA2S(1C4)-GlcNS6S-IdoA2S(1C4)-GlcNS6S	IdoA-GlcN	3	-63.5	-108.9	Xray
1BFB	4	ΔUA2S-GlcNS6S-IdoA2S(1C4)-GlcNS6S	ΔUA-GlcN	3	-78.8	-107.1	Xray
2HYU	4	ΔUA2S-GlcNS6S-IdoA2S(1C4)-GlcNS6S	ΔUA-GlcN	3	-62.2	-114.6	Xray
5E9C	4	ΔUA2S-GlcNS6S-IdoA-GlcNS6S	ΔUA-GlcN	3	-70.6	-112.1	Xray
1T8U	4	ΔUA2S-GlcNS6S-IdoA2S(2SO)-GlcNS6S	ΔUA-GlcN	3	-84	-113.5	Xray
3E7J	4	ΔUA-GlcNAc-GlcA-GlcNAc	ΔUA-GlcN	3	-45	-122.8	Xray
2GD4	5	GlcNS6S-GlcA-GlcNS3S6S-IdoA2S(2SO)-GlcNS6S	IdoA-GlcN	1	-67.5	-108.7	Xray
4X7R	5	GlcNS6S-GlcA-GlcNS3S6S-IdoA2S(2SO)-GlcNS6S	IdoA-GlcN	1	-65.2	-106.8	Xray
4R9W	5	GlcNS6S-GlcA-GlcNS3S6S-IdoA2S(2SO)-GlcNS6S	IdoA-GlcN	1	-76.9	-139.2	Xray

PDB	Length	Sequence	Linkage Type	Linkage Position	Φ	Ψ	Xray/NMR
3EVJ	5	GlcNS6S-GlcA-GlcNS3S6S-IdoA2S(2SO)-GlcNS6S	IdoA-GlcN	1	-67.9	-110.9	Xray
1GMN	5	IdoA2S(2SO)-GlcNS6S-IdoA2S(1C4)-GlcNS6S-IdoA2S(2SO)	IdoA-GlcN	2	-72.8	-95.5	Xray
2HYV	5	ΔUA2S-GlcNS6S-IdoA2S(1C4)-GlcNS6S-IdoA2S(1C4)	IdoA-GlcN	2	-120.2	-160.8	Xray
3DY0	5	IdoA(2SO)-GlcNS6S-IdoA2S(1C4)-GlcNS6S-IdoA(1C4)	IdoA-GlcN	2	-81.4	-105.9	Xray
7B8H	5	IdoA2S(1C4)-GlcNS6S-IdoA2S(1C4)-GlcNS6S-IdoA2S(1C4)	IdoA-GlcN	2	-80.1	-109.7	Xray
2GD4	5	GlcNS6S-GlcA-GlcNS3S6S-IdoA2S(2SO)-GlcNS6S	GlcA-GlcN	3	-84.3	-121	Xray
4X7R	5	GlcNS6S-GlcA-GlcNS3S6S-IdoA2S(2SO)-GlcNS6S	GlcA-GlcN	3	-94.6	-100.5	Xray
4R9W	5	GlcNS6S-GlcA-GlcNS3S6S-IdoA2S(2SO)-GlcNS6S	GlcA-GlcN	3	-70.3	-105.1	Xray
3EVJ	5	GlcNS6S-GlcA-GlcNS3S6S-IdoA2S(2SO)-GlcNS6S	GlcA-GlcN	3	-71.1	-122.7	Xray
1GMN	5	IdoA2S(2SO)-GlcNS6S-IdoA2S(1C4)-GlcNS6S-IdoA2S(2SO)	IdoA-GlcN	4	-79.6	-87.4	Xray
3DY0	5	IdoA(2SO)-GlcNS6S-IdoA2S(1C4)-GlcNS6S-IdoA(1C4)	IdoA-GlcN	4	-97.6	-85.2	Xray
7B8H	5	IdoA2S(1C4)-GlcNS6S-IdoA2S(1C4)-GlcNS6S-IdoA2S(1C4)	IdoA-GlcN	4	-72.9	-108.1	Xray
2HYV	5	ΔUA2S-GlcNS6S-IdoA2S(1C4)-GlcNS6S-IdoA2S(1C4)	ΔUA-GlcN	4	-62.1	-118.3	Xray
1BFC	6	ΔUA2S-GlcNS6S-IdoA2S(1C4)-GlcNS6S-IdoA2S(2SO)-GlcNS6S	IdoA-GlcN	1	-74.7	-96.3	Xray
1XT3	6	ΔUA2S-GlcNS6S-IdoA2S(1C4)-GlcNS6S-IdoA2S(2SO)-GlcNS6S	IdoA-GlcN	1	-65.1	-160.7	Xray
3MKP	6	IdoA2S(2SO)-GlcNS6S-IdoA2S(1C4)-GlcNS6S-IdoA2S(1C4)-GlcNS6S	IdoA-GlcN	1	-96.8	-135.4	Xray
3OJV	6	ΔUA2S-GlcNS6S-IdoA2S(1C4)-GlcNS6S-IdoA2S(1C4)-GlcNS6S	IdoA-GlcN	1	4.5	-99.1	Xray

PDB	Length	Sequence	Linkage Type	Linkage Position	Φ	Ψ	Xray/NMR
4AK2	6	IdoA2S(1C4)-GlcNS6S-IdoA2S(1C4)-GlcNS6S-IdoA2S(1C4)-GlcNS6S	IdoA-GlcN	1	-75.6	-124	Xray
4PXQ	6	ΔUA2S-GlcNS6S-IdoA2S(1C4)-GlcNS6S-IdoA2S(2SO)-GlcNS6S	IdoA-GlcN	1	-78.3	-113.2	Xray
1XMN	6	GlcNS6S-IdoA2S(1C4)-GlcNS6S-IdoA2S(1C4)-GlcNS6S-IdoA2S(2SO)	IdoA-GlcN	2	-100.8	-122.8	Xray
4C4N	6	GlcNS6S-IdoA2S(2SO)-GlcNS6S-IdoA2S(1C4)-GlcNS6S-IdoA2S(1C4)	IdoA-GlcN	2	-68.5	-107.1	Xray
3UAN	6	GlcNAc6S-GlcA-GlcNS6S-IdoA2S(1C4)-GlcNS6S-GlcA	IdoA-GlcN	2	-75.2	-106.8	Xray
4RDA	6	GlcNS6S-IdoA2S(1C4)-GlcNS6S-IdoA2S(1C4)-GlcNS6S-IdoA2S(1C4)	IdoA-GlcN	2	-65.2	-87.2	Xray
5T05	6	GlcNS-GlcA-GlcNS-IdoA2S(1C4)-GlcNS-GlcA	IdoA-GlcN	2	-77.5	-112.6	Xray
5T0A	6	GlcNS-GlcA-GlcNS-IdoA2S(1C4)-GlcNS-GlcA	IdoA-GlcN	2	-78.1	-108.2	Xray
5T03	6	GlcNS-GlcA-GlcNS-IdoA2S(1C4)-GlcNS-GlcA	IdoA-GlcN	2	-75.7	-116.4	Xray
1BFC	6	ΔUA2S-GlcNS6S-IdoA2S(1C4)-GlcNS6S-IdoA2S(2SO)-GlcNS6S	IdoA-GlcN	3	-63.4	-99.4	Xray
1XT3	6	ΔUA2S-GlcNS6S-IdoA2S(1C4)-GlcNS6S-IdoA2S(2SO)-GlcNS6S	IdoA-GlcN	3	-61.4	-130.3	Xray
3MKP	6	IdoA2S(2SO)-GlcNS6S-IdoA2S(1C4)-GlcNS6S-IdoA2S(1C4)-GlcNS6S	IdoA-GlcN	3	-74.1	-98.7	Xray
3OJV	6	ΔUA2S-GlcNS6S-IdoA2S(1C4)-GlcNS6S-IdoA2S(1C4)-GlcNS6S	IdoA-GlcN	3	-63	-88	Xray
4AK2	6	IdoA2S(1C4)-GlcNS6S-IdoA2S(1C4)-GlcNS6S-IdoA2S(1C4)-GlcNS6S	IdoA-GlcN	3	-78.9	-115.2	Xray
4PXQ	6	ΔUA2S-GlcNS6S-IdoA2S(1C4)-GlcNS6S-IdoA2S(2SO)-GlcNS6S	IdoA-GlcN	3	-76.1	-121.9	Xray
3UAN	6	GlcNAc6S-GlcA-GlcNS6S-IdoA2S(1C4)-GlcNS6S-GlcA	GlcA-GlcN	4	-84.5	-103.3	Xray

PDB	Length	Sequence	Linkage Type	Linkage Position	Φ	Ψ	Xray/NMR
5T05	6	GlcNS-GlcA-GlcNS-IdoA2S(1C4)-GlcNS-GlcA	GlcA-GlcN	4	-81.8	-100.5	Xray
5T0A	6	GlcNS-GlcA-GlcNS-IdoA2S(1C4)-GlcNS-GlcA	GlcA-GlcN	4	-77.6	-101.5	Xray
5T03	6	GlcNS-GlcA-GlcNS-IdoA2S(1C4)-GlcNS-GlcA	GlcA-GlcN	4	-82.3	-100.6	Xray
1XMN	6	GlcNS6S-IdoA2S(1C4)-GlcNS6S-IdoA2S(1C4)-GlcNS6S-IdoA2S(2SO)	IdoA-GlcN	4	-79.2	-91.4	Xray
4C4N	6	GlcNS6S-IdoA2S(2SO)-GlcNS6S-IdoA2S(1C4)-GlcNS6S-IdoA2S(1C4)	IdoA-GlcN	4	-70	-99.5	Xray
4RDA	6	GlcNS6S-IdoA2S(1C4)-GlcNS6S-IdoA2S(1C4)-GlcNS6S-IdoA2S(1C4)	IdoA-GlcN	4	-71.7	-113.6	Xray
3MKP	6	IdoA2S(2SO)-GlcNS6S-IdoA2S(1C4)-GlcNS6S-IdoA2S(1C4)-GlcNS6S	IdoA-GlcN	5	-73.9	-102.4	Xray
4AK2	6	IdoA2S(1C4)-GlcNS6S-IdoA2S(1C4)-GlcNS6S-IdoA2S(1C4)-GlcNS6S	IdoA-GlcN	5	-73.4	-118.4	Xray
1BFC	6	ΔUA2S-GlcNS6S-IdoA2S(1C4)-GlcNS6S-IdoA2S(2SO)-GlcNS6S	ΔUA-GlcN	5	-66.6	-115.7	Xray
1XT3	6	ΔUA2S-GlcNS6S-IdoA2S(1C4)-GlcNS6S-IdoA2S(2SO)-GlcNS6S	ΔUA-GlcN	5	-65.6	-129.8	Xray
3OJV	6	ΔUA2S-GlcNS6S-IdoA2S(1C4)-GlcNS6S-IdoA2S(1C4)-GlcNS6S	ΔUA-GlcN	5	99.9	68.7	Xray
4PXQ	6	ΔUA2S-GlcNS6S-IdoA2S(1C4)-GlcNS6S-IdoA2S(2SO)-GlcNS6S	ΔUA-GlcN	5	-62.3	-105.4	Xray
5UE2	8	IdoA2S(1C4)-GlcNS6S-IdoA2S(1C4)-GlcNS6S-IdoA2S(1C4)-GlcNS6S-IdoA2S(1C4)-GlcNS6S	IdoA-GlcN	1	-91.6	-161.3	NMR
5UE5	8	IdoA2S(1C4)-GlcNS6S-IdoA2S(1C4)-GlcNS6S-IdoA2S(1C4)-GlcNS6S-IdoA2S(1C4)-GlcNS6S	IdoA-GlcN	1	-158.8	-158.1	NMR

PDB	Length	Sequence	Linkage Type	Linkage Position	Φ	Ψ	Xray/NMR
7B8I	8	GlcNS6S-IdoA2S(1C4)-GlcNS6S-IdoA2S(1C4)-GlcNS6S-IdoA2S(1C4)-GlcNS6S-IdoA2S(1C4)	IdoA-GlcN	2	-110.2	-103.7	Xray
3INA	8	GlcNS6S-IdoA2S(2SO)-GlcNS3S6S-IdoA2S(1C4)-GlcNS6S-IdoA2S(1C4)-GlcNS6S-IdoA2S(1C4)	IdoA-GlcN	2	-48	-98.8	Xray
5UE2	8	IdoA2S(1C4)-GlcNS6S-IdoA2S(1C4)-GlcNS6S-IdoA2S(1C4)-GlcNS6S-IdoA2S(1C4)-GlcNS6S	IdoA-GlcN	3	-64	-94.8	NMR
5UE5	8	IdoA2S(1C4)-GlcNS6S-IdoA2S(1C4)-GlcNS6S-IdoA2S(1C4)-GlcNS6S-IdoA2S(1C4)-GlcNS6S	IdoA-GlcN	3	-88.3	-168.1	NMR
7B8I	8	GlcNS6S-IdoA2S(1C4)-GlcNS6S-IdoA2S(1C4)-GlcNS6S-IdoA2S(1C4)-GlcNS6S-IdoA2S(1C4)	IdoA-GlcN	4	-93.7	-117	Xray
3INA	8	GlcNS6S-IdoA2S(2SO)-GlcNS3S6S-IdoA2S(1C4)-GlcNS6S-IdoA2S(1C4)-GlcNS6S-IdoA2S(1C4)	IdoA-GlcN	4	-133.17	63.53	Xray
5UE2	8	IdoA2S(1C4)-GlcNS6S-IdoA2S(1C4)-GlcNS6S-IdoA2S(1C4)-GlcNS6S-IdoA2S(1C4)-GlcNS6S	IdoA-GlcN	5	-80.2	-158.9	NMR
5UE5	8	IdoA2S(1C4)-GlcNS6S-IdoA2S(1C4)-GlcNS6S-IdoA2S(1C4)-GlcNS6S-IdoA2S(1C4)-GlcNS6S	IdoA-GlcN	5	-126	-142.7	NMR
7B8I	8	GlcNS6S-IdoA2S(1C4)-GlcNS6S-IdoA2S(1C4)-GlcNS6S-IdoA2S(1C4)-GlcNS6S-IdoA2S(1C4)	IdoA-GlcN	6	-112.5	80.4	Xray
3INA	8	GlcNS6S-IdoA2S(2SO)-GlcNS3S6S-IdoA2S(1C4)-GlcNS6S-IdoA2S(1C4)-GlcNS6S-IdoA2S(1C4)	IdoA-GlcN	6	-64.04	-100.97	Xray

PDB	Length	Sequence	Linkage Type	Linkage Position	Φ	Ψ	Xray/NMR
5UE2	8	IdoA2S(1C4)-GlcNS6S-IdoA2S(1C4)-GlcNS6S-IdoA2S(1C4)-GlcNS6S-IdoA2S(1C4)-GlcNS6S	IdoA-GlcN	7	-70.3	-150.1	NMR
5UE5	8	IdoA2S(1C4)-GlcNS6S-IdoA2S(1C4)-GlcNS6S-IdoA2S(1C4)-GlcNS6S-IdoA2S(1C4)-GlcNS6S	IdoA-GlcN	7	-83.5	-159.6	NMR
1GMO	9	GlcNS6S-IdoA2S(2SO)-GlcNS6S-IdoA2S(1C4)-GlcNS6S-IdoA2S(1C4)-GlcNS6S-IdoA2S(2SO)-GlcNS6S	IdoA-GlcN	1	-87.5	-113.3	Xray
1GMO	9	GlcNS6S-IdoA2S(2SO)-GlcNS6S-IdoA2S(1C4)-GlcNS6S-IdoA2S(1C4)-GlcNS6S-IdoA2S(2SO)-GlcNS6S	IdoA-GlcN	3	-101.3	-124.5	Xray
1GMO	9	GlcNS6S-IdoA2S(2SO)-GlcNS6S-IdoA2S(1C4)-GlcNS6S-IdoA2S(1C4)-GlcNS6S-IdoA2S(2SO)-GlcNS6S	IdoA-GlcN	5	-75	-102.7	Xray
1GMO	9	GlcNS6S-IdoA2S(2SO)-GlcNS6S-IdoA2S(1C4)-GlcNS6S-IdoA2S(1C4)-GlcNS6S-IdoA2S(2SO)-GlcNS6S	IdoA-GlcN	7	-93.6	-82.6	Xray
1E0O	10	IdoA2S(2SO)-GlcNS6S-IdoA2S(2SO)-GlcNS6S-IdoA2S(2SO)-GlcNS6S-IdoA2S(2SO)-GlcNS6S-IdoA2S(1C4)-GlcNS6S	IdoA-GlcN	1	-79.6	-107.6	Xray
1E0O	10	IdoA2S(2SO)-GlcNS6S-IdoA2S(2SO)-GlcNS6S-IdoA2S(2SO)-GlcNS6S-IdoA2S(2SO)-GlcNS6S-IdoA2S(1C4)-GlcNS6S	IdoA-GlcN	3	-62.1	-164.8	Xray
1E0O	10	IdoA2S(2SO)-GlcNS6S-IdoA2S(2SO)-GlcNS6S-IdoA2S(2SO)-GlcNS6S-IdoA2S(2SO)-GlcNS6S-IdoA2S(1C4)-GlcNS6S	IdoA-GlcN	5	-76.4	-103.2	Xray

PDB	Length	Sequence	Linkage Type	Linkage Position	Φ	Ψ	Xray/NMR
1E0O	10	IdoA2S(2SO)-GlcNS6S-IdoA2S(2SO)-GlcNS6S-IdoA2S(2SO)-GlcNS6S-IdoA2S(2SO)-GlcNS6S-IdoA2S(1C4)-GlcNS6S	IdoA-GlcN	7	-64.8	-116.5	Xray
1E0O	10	IdoA2S(2SO)-GlcNS6S-IdoA2S(2SO)-GlcNS6S-IdoA2S(2SO)-GlcNS6S-IdoA2S(2SO)-GlcNS6S-IdoA2S(1C4)-GlcNS6S	IdoA-GlcN	9	-106.1	-131	Xray
1HPN	12	IdoA(2SO)-GlcNS6S(6x)	IdoA-GlcN	1	-55.3	-107.5	NMR
1HPN	12	IdoA(2SO)-GlcNS6S(6x)	IdoA-GlcN	3	-55.4	-107.5	NMR
1HPN	12	IdoA(2SO)-GlcNS6S(6x)	IdoA-GlcN	5	-55.4	-107.6	NMR
1HPN	12	IdoA(2SO)-GlcNS6S(6x)	IdoA-GlcN	7	-55.3	-107.5	NMR
1HPN	12	IdoA(2SO)-GlcNS6S(6x)	IdoA-GlcN	9	-55.3	-107.5	NMR
1HPN	12	IdoA(2SO)-GlcNS6S(6x)	IdoA-GlcN	11	-55.2	-107.7	NMR

Table S3. Phi and psi corresponding to different glycosidic linkages of Hp/HS complexes with diverse proteins obtained from the protein data bank (PDB). Shown below are Φ and Ψ values for amine – acid linkages (GlcN→UA).

PDB	Length	Sequence	Linkage Type	Linkage Position	Φ	Ψ	Xray/NMR
3E7J	4	ΔUA-GlcNAc-GlcA-GlcNAc	GlcN-GlcA	2	84.9	-145.6	Xray
2GD4	5	GlcNS6S-GlcA-GlcNS3S6S-IdoA2S(2SO)-GlcNS6S	GlcN-GlcA	4	101.5	-158	Xray
4X7R	5	GlcNS6S-GlcA-GlcNS3S6S-IdoA2S(2SO)-GlcNS6S	GlcN-GlcA	4	96.5	-128.2	Xray
4R9W	5	GlcNS6S-GlcA-GlcNS3S6S-IdoA2S(2SO)-GlcNS6S	GlcN-GlcA	4	69.5	-155.4	Xray
3EVJ	5	GlcNS6S-GlcA-GlcNS3S6S-IdoA2S(2SO)-GlcNS6S	GlcN-GlcA	4	100.5	-167.2	Xray
3UAN	6	GlcNAc6S-GlcA-GlcNS6S-IdoA2S(1C4)-GlcNS6S-GlcA	GlcN-GlcA	1	72.7	-144	Xray
3UAN	6	GlcNAc6S-GlcA-GlcNS6S-IdoA2S(1C4)-GlcNS6S-GlcA	GlcN-GlcA	5	90.3	-156.1	Xray
5T05	6	GlcNS-GlcA-GlcNS-IdoA2S(1C4)-GlcNS-GlcA	GlcN-GlcA	1	111.6	-110.9	Xray
5T05	6	GlcNS-GlcA-GlcNS-IdoA2S(1C4)-GlcNS-GlcA	GlcN-GlcA	5	82.6	-146.9	Xray
5T0A	6	GlcNS-GlcA-GlcNS-IdoA2S(1C4)-GlcNS-GlcA	GlcN-GlcA	1	108.7	-106.2	Xray
5T0A	6	GlcNS-GlcA-GlcNS-IdoA2S(1C4)-GlcNS-GlcA	GlcN-GlcA	5	81.8	-145.9	Xray
5T03	6	GlcNS-GlcA-GlcNS-IdoA2S(1C4)-GlcNS-GlcA	GlcN-GlcA	1	102.5	-100.7	Xray
5T03	6	GlcNS-GlcA-GlcNS-IdoA2S(1C4)-GlcNS-GlcA	GlcN-GlcA	5	79.5	-145.9	Xray
5DNF	3	GlcNS6S-IdoA2S(1C4)-GlcNS6S	GlcN-IdoA	2	84.7	-128	Xray
1BFB	4	ΔUA2S-GlcNS6S-IdoA2S(1C4)-GlcNS6S	GlcN-IdoA	2	94.5	-129.8	Xray
2HYU	4	ΔUA2S-GlcNS6S-IdoA2S(1C4)-GlcNS6S	GlcN-IdoA	2	80.9	-141.5	Xray
2VRA	4	IdoA2S(2SO)-GlcNS6S-IdoA2S(1C4)-GlcNS6S	GlcN-IdoA	2	89.8	-97.4	Xray
3QMK	4	IdoA2S(1C4)-GlcNS6S-IdoA2S(1C4)-GlcNS6S	GlcN-IdoA	2	81.1	-145.5	Xray
5E9C	4	ΔUA2S-GlcNS6S-IdoA-GlcNS6S	GlcN-IdoA	2	50	-134.8	Xray

PDB	Length	Sequence	Linkage Type	Linkage Position	Φ	Ψ	Xray/NMR
1T8U	4	ΔUA2S-GlcNS6S-IdoA2S(2SO)-GlcNS6S	GlcN-IdoA	2	55.3	-176.1	Xray
1GMN	5	IdoA2S(2SO)-GlcNS6S-IdoA2S(1C4)-GlcNS6S-IdoA2S(2SO)	GlcN-IdoA	1	78.4	-61.3	Xray
1GMN	5	IdoA2S(2SO)-GlcNS6S-IdoA2S(1C4)-GlcNS6S-IdoA2S(2SO)	GlcN-IdoA	3	82.6	-132.2	Xray
2HYV	5	ΔUA2S-GlcNS6S-IdoA2S(1C4)-GlcNS6S-IdoA2S(1C4)	GlcN-IdoA	1	74.1	-141.2	Xray
2HYV	5	ΔUA2S-GlcNS6S-IdoA2S(1C4)-GlcNS6S-IdoA2S(1C4)	GlcN-IdoA	3	81.5	-142.3	Xray
3DY0	5	IdoA(2SO)-GlcNS6S-IdoA2S(1C4)-GlcNS6S-IdoA(1C4)	GlcN-IdoA	1	77.4	-166	Xray
3DY0	5	IdoA(2SO)-GlcNS6S-IdoA2S(1C4)-GlcNS6S-IdoA(1C4)	GlcN-IdoA	3	75.4	-134.4	Xray
7B8H	5	IdoA2S(1C4)-GlcNS6S-IdoA2S(1C4)-GlcNS6S-IdoA2S(1C4)	GlcN-IdoA	1	90	-126.4	Xray
7B8H	5	IdoA2S(1C4)-GlcNS6S-IdoA2S(1C4)-GlcNS6S-IdoA2S(1C4)	GlcN-IdoA	3	101.2	-121.6	Xray
2GD4	5	GlcNS6S-GlcA-GlcNS3S6S-IdoA2S(2SO)-GlcNS6S	GlcN-IdoA	2	62.5	-156.2	Xray
4X7R	5	GlcNS6S-GlcA-GlcNS3S6S-IdoA2S(2SO)-GlcNS6S	GlcN-IdoA	2	73.2	-151.9	Xray
4R9W	5	GlcNS6S-GlcA-GlcNS3S6S-IdoA2S(2SO)-GlcNS6S	GlcN-IdoA	2	71	-132	Xray
3EVJ	5	GlcNS6S-GlcA-GlcNS3S6S-IdoA2S(2SO)-GlcNS6S	GlcN-IdoA	2	58.7	-160.9	Xray
1BFC	6	ΔUA2S-GlcNS6S-IdoA2S(1C4)-GlcNS6S-IdoA2S(2SO)-GlcNS6S	GlcN-IdoA	2	83.1	-158.2	Xray
1BFC	6	ΔUA2S-GlcNS6S-IdoA2S(1C4)-GlcNS6S-IdoA2S(2SO)-GlcNS6S	GlcN-IdoA	4	93.7	-125.6	Xray
1XMN	6	GlcNS6S-IdoA2S(1C4)-GlcNS6S-IdoA2S(1C4)-GlcNS6S-IdoA2S(2SO)	GlcN-IdoA	1	147.5	-104.2	Xray
1XMN	6	GlcNS6S-IdoA2S(1C4)-GlcNS6S-IdoA2S(1C4)-GlcNS6S-IdoA2S(2SO)	GlcN-IdoA	3	68.6	-145.7	Xray

PDB	Length	Sequence	Linkage Type	Linkage Position	Φ	Ψ	Xray/NMR
1XMN	6	GlcNS6S-IdoA2S(1C4)-GlcNS6S-IdoA2S(1C4)-GlcNS6S-IdoA2S(2SO)	GlcN-IdoA	5	58.7	-159.5	Xray
1XT3	6	ΔUA2S-GlcNS6S-IdoA2S(1C4)-GlcNS6S-IdoA2S(2SO)-GlcNS6S	GlcN-IdoA	2	130.5	-122.4	Xray
1XT3	6	ΔUA2S-GlcNS6S-IdoA2S(1C4)-GlcNS6S-IdoA2S(2SO)-GlcNS6S	GlcN-IdoA	4	115.3	-155.2	Xray
3MKP	6	IdoA2S(2SO)-GlcNS6S-IdoA2S(1C4)-GlcNS6S-IdoA2S(1C4)-GlcNS6S	GlcN-IdoA	2	85.8	-137.2	Xray
3MKP	6	IdoA2S(2SO)-GlcNS6S-IdoA2S(1C4)-GlcNS6S-IdoA2S(1C4)-GlcNS6S	GlcN-IdoA	4	100.8	-132.3	Xray
3OJV	6	ΔUA2S-GlcNS6S-IdoA2S(1C4)-GlcNS6S-IdoA2S(1C4)-GlcNS6S	GlcN-IdoA	2	89.9	-158.5	Xray
3OJV	6	ΔUA2S-GlcNS6S-IdoA2S(1C4)-GlcNS6S-IdoA2S(1C4)-GlcNS6S	GlcN-IdoA	4	77	-144.6	Xray
4AK2	6	IdoA2S(1C4)-GlcNS6S-IdoA2S(1C4)-GlcNS6S-IdoA2S(1C4)-GlcNS6S	GlcN-IdoA	2	83.2	-143.5	Xray
4AK2	6	IdoA2S(1C4)-GlcNS6S-IdoA2S(1C4)-GlcNS6S-IdoA2S(1C4)-GlcNS6S	GlcN-IdoA	4	95.2	-102.2	Xray
4C4N	6	GlcNS6S-IdoA2S(2SO)-GlcNS6S-IdoA2S(1C4)-GlcNS6S-IdoA2S(1C4)	GlcN-IdoA	1	68.8	-149.9	Xray
4C4N	6	GlcNS6S-IdoA2S(2SO)-GlcNS6S-IdoA2S(1C4)-GlcNS6S-IdoA2S(1C4)	GlcN-IdoA	3	86.4	-149.4	Xray
4C4N	6	GlcNS6S-IdoA2S(2SO)-GlcNS6S-IdoA2S(1C4)-GlcNS6S-IdoA2S(1C4)	GlcN-IdoA	5	128.5	-112.1	Xray
4PXQ	6	ΔUA2S-GlcNS6S-IdoA2S(1C4)-GlcNS6S-IdoA2S(2SO)-GlcNS6S	GlcN-IdoA	2	42.9	-155.3	Xray
4PXQ	6	ΔUA2S-GlcNS6S-IdoA2S(1C4)-GlcNS6S-IdoA2S(2SO)-GlcNS6S	GlcN-IdoA	4	68.2	-145.4	Xray
3UAN	6	GlcNAc6S-GlcA-GlcNS6S-IdoA2S(1C4)-GlcNS6S-GlcA	GlcN-IdoA	3	60.8	-153.8	Xray

PDB	Length	Sequence	Linkage Type	Linkage Position	Φ	Ψ	Xray/NMR
4RDA	6	GlcNS6S-IdoA2S(1C4)-GlcNS6S-IdoA2S(1C4)-GlcNS6S-IdoA2S(1C4)	GlcN-IdoA	1	41.8	-166.3	Xray
4RDA	6	GlcNS6S-IdoA2S(1C4)-GlcNS6S-IdoA2S(1C4)-GlcNS6S-IdoA2S(1C4)	GlcN-IdoA	3	27.1	-167.2	Xray
4RDA	6	GlcNS6S-IdoA2S(1C4)-GlcNS6S-IdoA2S(1C4)-GlcNS6S-IdoA2S(1C4)	GlcN-IdoA	5	74.2	-147	Xray
5T05	6	GlcNS-GlcA-GlcNS-IdoA2S(1C4)-GlcNS-GlcA	GlcN-IdoA	3	80.2	-142.7	Xray
5T0A	6	GlcNS-GlcA-GlcNS-IdoA2S(1C4)-GlcNS-GlcA	GlcN-IdoA	3	79.2	-143.1	Xray
5T03	6	GlcNS-GlcA-GlcNS-IdoA2S(1C4)-GlcNS-GlcA	GlcN-IdoA	3	70.5	-139	Xray
5UE2	8	IdoA2S(1C4)-GlcNS6S-IdoA2S(1C4)-GlcNS6S-IdoA2S(1C4)-GlcNS6S-IdoA2S(1C4)-GlcNS6S	GlcN-IdoA	2	85.2	-149.5	NMR
5UE2	8	IdoA2S(1C4)-GlcNS6S-IdoA2S(1C4)-GlcNS6S-IdoA2S(1C4)-GlcNS6S-IdoA2S(1C4)-GlcNS6S	GlcN-IdoA	4	89.2	-153.2	NMR
5UE2	8	IdoA2S(1C4)-GlcNS6S-IdoA2S(1C4)-GlcNS6S-IdoA2S(1C4)-GlcNS6S-IdoA2S(1C4)-GlcNS6S	GlcN-IdoA	6	80.3	-147.8	NMR
5UE5	8	IdoA2S(1C4)-GlcNS6S-IdoA2S(1C4)-GlcNS6S-IdoA2S(1C4)-GlcNS6S-IdoA2S(1C4)-GlcNS6S	GlcN-IdoA	2	123.1	-115.6	NMR
5UE5	8	IdoA2S(1C4)-GlcNS6S-IdoA2S(1C4)-GlcNS6S-IdoA2S(1C4)-GlcNS6S-IdoA2S(1C4)-GlcNS6S	GlcN-IdoA	4	79.1	-146.8	NMR
5UE5	8	IdoA2S(1C4)-GlcNS6S-IdoA2S(1C4)-GlcNS6S-IdoA2S(1C4)-GlcNS6S-IdoA2S(1C4)-GlcNS6S	GlcN-IdoA	6	97	-149.6	NMR

PDB	Length	Sequence	Linkage Type	Linkage Position	Φ	Ψ	Xray/NMR
7B8I	8	GlcNS6S-IdoA2S(1C4)-GlcNS6S-IdoA2S(1C4)-GlcNS6S-IdoA2S(1C4)-GlcNS6S-IdoA2S(1C4)	GlcN-IdoA	1	50.1	-170.4	Xray
7B8I	8	GlcNS6S-IdoA2S(1C4)-GlcNS6S-IdoA2S(1C4)-GlcNS6S-IdoA2S(1C4)-GlcNS6S-IdoA2S(1C4)	GlcN-IdoA	3	72.4	-152.2	Xray
7B8I	8	GlcNS6S-IdoA2S(1C4)-GlcNS6S-IdoA2S(1C4)-GlcNS6S-IdoA2S(1C4)-GlcNS6S-IdoA2S(1C4)	GlcN-IdoA	5	113.6	-142.1	Xray
7B8I	8	GlcNS6S-IdoA2S(1C4)-GlcNS6S-IdoA2S(1C4)-GlcNS6S-IdoA2S(1C4)-GlcNS6S-IdoA2S(1C4)	GlcN-IdoA	7	-52.4	-144.8	Xray
3INA	8	GlcNS6S-IdoA2S(2SO)-GlcNS3S6S-IdoA2S(1C4)-GlcNS6S-IdoA2S(1C4)-GlcNS6S-IdoA2S(1C4)	GlcN-IdoA	1	82.84	-149.76	Xray
3INA	8	GlcNS6S-IdoA2S(2SO)-GlcNS3S6S-IdoA2S(1C4)-GlcNS6S-IdoA2S(1C4)-GlcNS6S-IdoA2S(1C4)	GlcN-IdoA	3	76.91	-145.91	Xray
3INA	8	GlcNS6S-IdoA2S(2SO)-GlcNS3S6S-IdoA2S(1C4)-GlcNS6S-IdoA2S(1C4)-GlcNS6S-IdoA2S(1C4)	GlcN-IdoA	5	91.1	-151.28	Xray
3INA	8	GlcNS6S-IdoA2S(2SO)-GlcNS3S6S-IdoA2S(1C4)-GlcNS6S-IdoA2S(1C4)-GlcNS6S-IdoA2S(1C4)	GlcN-IdoA	7	98.72	-134.84	Xray
1GMO	9	GlcNS6S-IdoA2S(2SO)-GlcNS6S-IdoA2S(1C4)-GlcNS6S-IdoA2S(1C4)-GlcNS6S-IdoA2S(2SO)-GlcNS6S	GlcN-IdoA	2	54	-142.5	Xray
1GMO	9	GlcNS6S-IdoA2S(2SO)-GlcNS6S-IdoA2S(1C4)-GlcNS6S-IdoA2S(1C4)-GlcNS6S-IdoA2S(2SO)-GlcNS6S	GlcN-IdoA	4	91.7	-144.3	Xray

PDB	Length	Sequence	Linkage Type	Linkage Position	Φ	Ψ	Xray/NMR
1GMO	9	GlcNS6S-IdoA2S(2SO)-GlcNS6S-IdoA2S(1C4)-GlcNS6S-IdoA2S(1C4)-GlcNS6S-IdoA2S(2SO)-GlcNS6S	GlcN-IdoA	6	79.5	-129.4	Xray
1GMO	9	GlcNS6S-IdoA2S(2SO)-GlcNS6S-IdoA2S(1C4)-GlcNS6S-IdoA2S(1C4)-GlcNS6S-IdoA2S(2SO)-GlcNS6S	GlcN-IdoA	8	76	-140.4	Xray
1E0O	10	IdoA2S(2SO)-GlcNS6S-IdoA2S(2SO)-GlcNS6S-IdoA2S(2SO)-GlcNS6S-IdoA2S(2SO)-GlcNS6S-IdoA2S(1C4)-GlcNS6S	GlcN-IdoA	2	78.6	-140.4	Xray
1E0O	10	IdoA2S(2SO)-GlcNS6S-IdoA2S(2SO)-GlcNS6S-IdoA2S(2SO)-GlcNS6S-IdoA2S(2SO)-GlcNS6S-IdoA2S(1C4)-GlcNS6S	GlcN-IdoA	4	141.4	-142.8	Xray
1E0O	10	IdoA2S(2SO)-GlcNS6S-IdoA2S(2SO)-GlcNS6S-IdoA2S(2SO)-GlcNS6S-IdoA2S(2SO)-GlcNS6S-IdoA2S(1C4)-GlcNS6S	GlcN-IdoA	6	117.6	-134.8	Xray
1E0O	10	IdoA2S(2SO)-GlcNS6S-IdoA2S(2SO)-GlcNS6S-IdoA2S(2SO)-GlcNS6S-IdoA2S(2SO)-GlcNS6S-IdoA2S(1C4)-GlcNS6S	GlcN-IdoA	8	134.7	-151.1	Xray
1HPN	12	IdoA(2SO)-GlcNS6S(6x)	GlcN-IdoA	2	108.5	-157.7	NMR
1HPN	12	IdoA(2SO)-GlcNS6S(6x)	GlcN-IdoA	4	108.5	-157.7	NMR
1HPN	12	IdoA(2SO)-GlcNS6S(6x)	GlcN-IdoA	6	108.4	-157.8	NMR
1HPN	12	IdoA(2SO)-GlcNS6S(6x)	GlcN-IdoA	8	108.5	-157.7	NMR
1HPN	12	IdoA(2SO)-GlcNS6S(6x)	GlcN-IdoA	10	108.4	-157.7	NMR

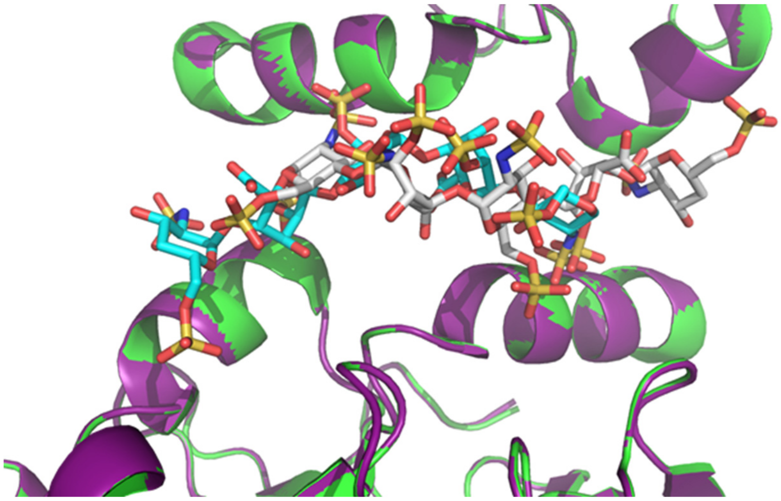

Figure S3. Comparative structures of two heparin oligosaccharides bound to thrombin in the 1XMN (thrombin-Hp complex, protein in purple and ligand in cyan) and 1TB6 (AT-Thrombin-HP complex, protein in green and ligand in orange,) crystal structure used in this study. The oligomers, although very similar, bind in different geometries.

Table S4. Structures of heparan sulfate oligosaccharides present in co-crystal structures.

PDB	Chain Length	Sequence	Protein(s)	Resolution (Å)
1U4L	2	ΔUA2S-GlcNS6S	RANTES	2.00
1U4M	2	ΔUA2S-GlcNS	RANTES	2.00
3B9F	2	IdoA2S(^2S_O)-GlcNS	Thrombin and PCI	1.60
5DNF	3	GlcNS6S-IdoA2S($^1C_4$)-GlcNS6S	RANTES	2.55
2HYU	4	ΔUA2S-GlcNS6S-IdoA2S($^1C_4$)-GlcNS6S	Annexin A2	1.86
2VRA	4	IdoA2S(^2S_O)-GlcNS6S-IdoA2S($^1C_4$)-GlcNS6S	Robo	3.20
6LJL	4	ΔUA2S-GlcNS6S-IdoA2S($^1C_4$)-GlcNS6S	Hep-Y390A/H555A	1.73
3EVJ	5	GlcNS6S-GlcA-GlcNS3S6S-IdoA2S(^2S_O)-GlcNS6S	AT	3.00
1TB6	5	GlcNS6S-GlcA-GlcNS3S6S-IdoA2S(^2S_O)-GlcNS6S	AT	2.50
4R9W	5	GlcNS6S-GlcA-GlcNS3S6S-IdoA2S(^2S_O)-GlcNS6S	PF4	2.50
2HYV	5	ΔUA2S-GlcNS6S-IdoA2S($^1C_4$)-GlcNS6S-IdoA2S($^1C_4$)	Annexin 2	1.42
4AK2	6	IdoA2S($^1C_4$)-GlcNS6S-IdoA2S($^1C_4$)-GlcNS6S-	BT4661	1.35
1XMN	6	GlcNS6S-IdoA2S($^1C_4$)-GlcNS6S-IdoA2S($^1C_4$)-GlcNS6S-	Thrombin	1.85
3UAN	6	GlcNAc6S-GlcA-GlcNS6S-IdoA2S($^1C_4$)-GlcNS6S-GlcA	3OST1	1.84
4C4N	6	IdoA2S($^1C_4$)-GlcNS6S-IdoA2S($^1C_4$)-GlcNS6S-	Hedgehog	2.36
3INA	8	GlcNS6S-IdoA2S(^2S_O)-GlcNS3S6S-IdoA2S($^1C_4$)-<br>GlcNS6S-IdoA2S($^1C_4$)-GlcNS6S-IdoA2S($^1C_4$)	H151A Heparinase 1	1.90
7B8I	8	GlcNS6S-IdoA2S($^1C_4$)-GlcNS6S-IdoA2S($^1C_4$)-GlcNS6S-<br>IdoA2S($^1C_4$)-GlcNS6S-IdoA2S($^1C_4$)	Protein kinase CK2 catalytic subunit	2.55
1E0O	10	IdoA2S(^2S_O)-GlcNS6S-IdoA2S(^2S_O)-GlcNS6S-<br>IdoA2S(^2S_O)-GlcNS6S-IdoA2S(^2S_O)-GlcNS6S-	FGF1 and FGFR2	2.80

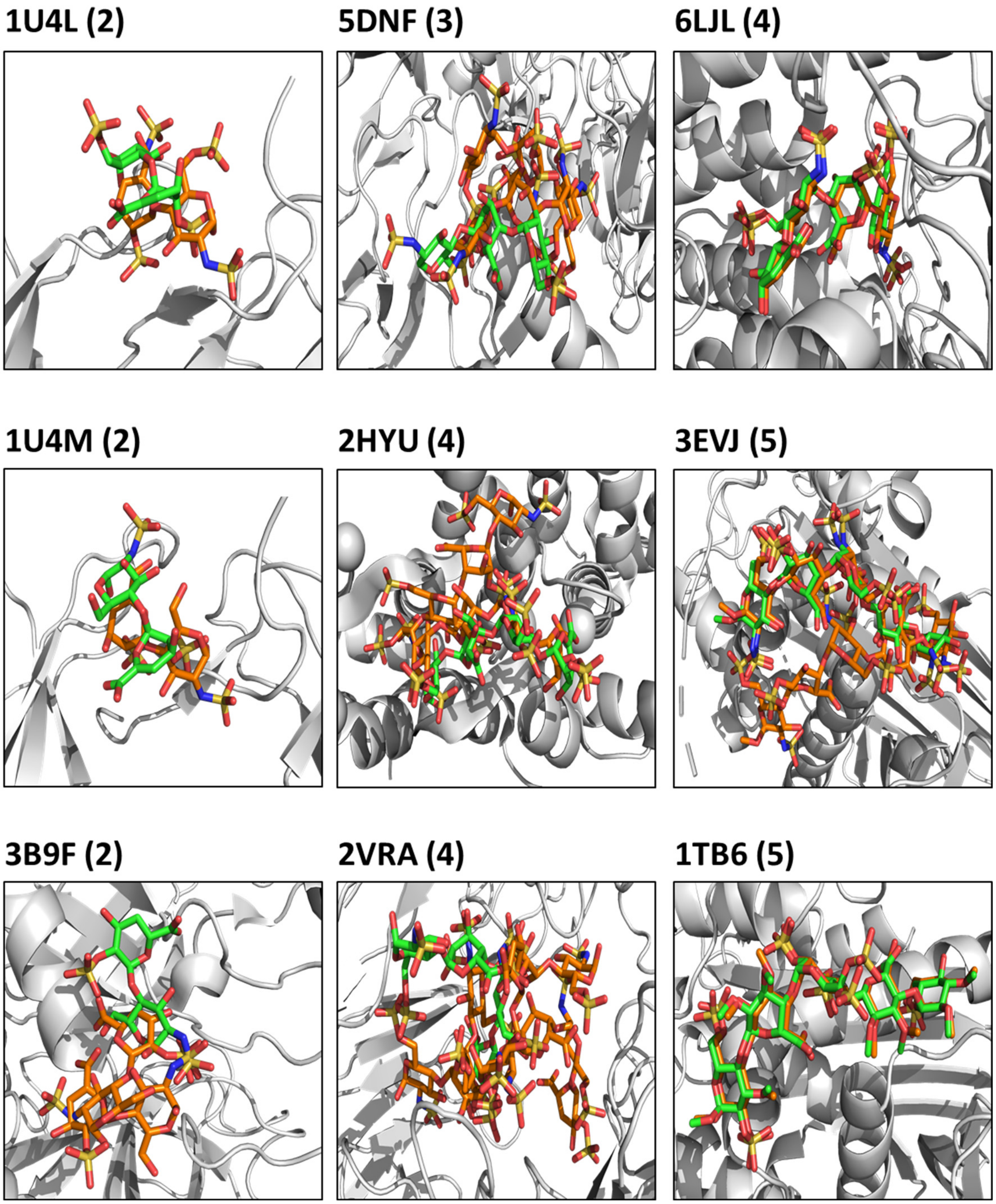

Figure S4 (Part I). Recapitulation of the native pose using the rigid docking protocol. Each sequence was redocked back into the crystal structure in triplicate using 100 GA runs, each being allowed 100,000 genetic operations. The top two poses from each replicate experiment were selected and compared by calculating the $RMSD_{AVERAGE}$, $RMSD_{LOWEST}$ and $RMSD_{INTRAPOSE}$ (see **Figure 2** for definitions). Representative docking poses from the top 6 obtained for all 18 protein–Hp/HS oligosaccharide complexes are shown here. Redundant poses are not presented for clarity. Native poses in each both are shown in green, while docked poses are in orange.

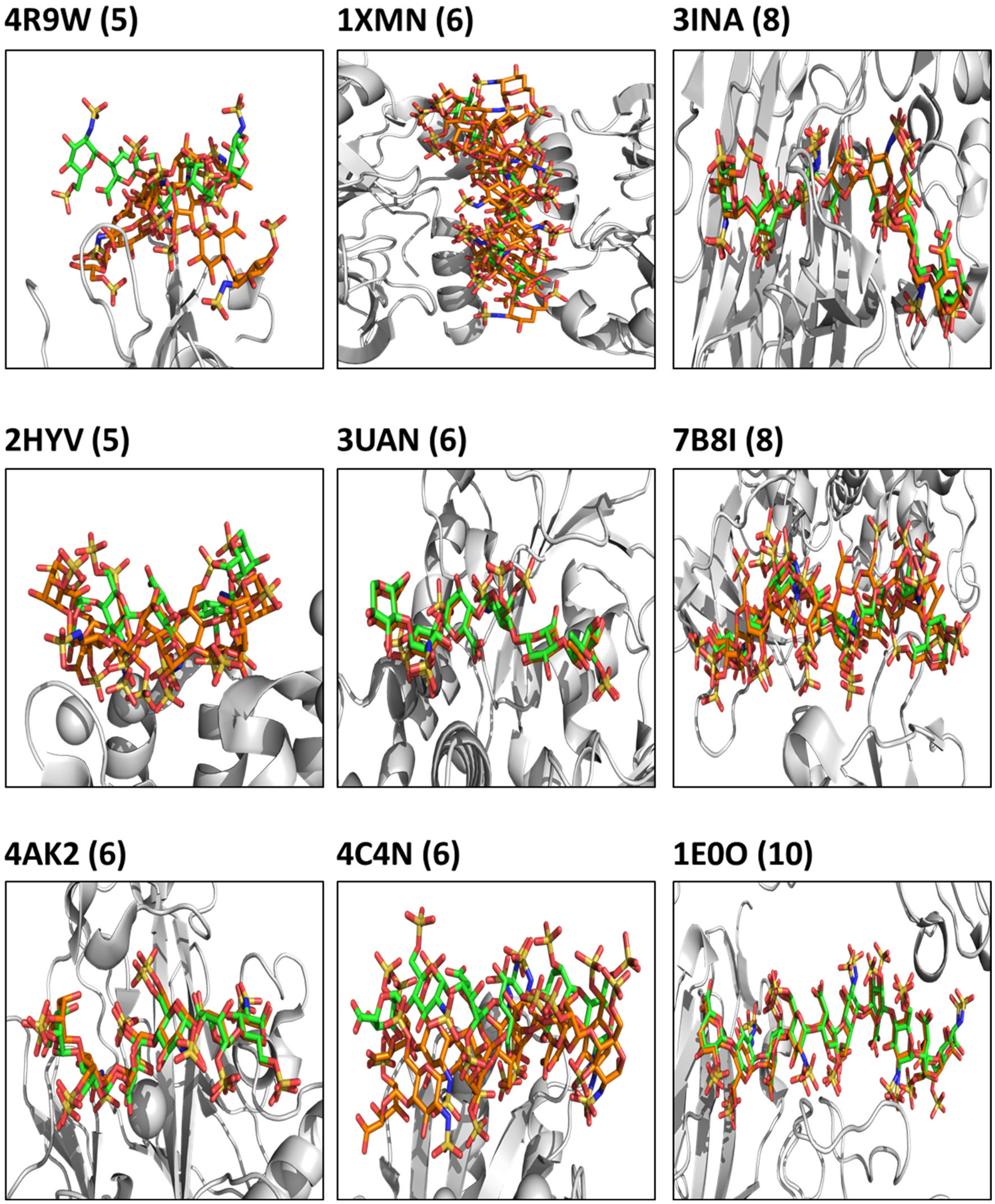

Figure S4 (Part II). Recapitulation of the native pose using the rigid docking protocol. Each sequence was redocked back into the crystal structure in triplicate using 100 GA runs, each being allowed 100,000 genetic operations. The top two poses from each replicate experiment were selected and compared by calculating the $RMSD_{AVERAGE}$, $RMSD_{LOWEST}$ and $RMSD_{INTRAPOSE}$ (see **Figure 2** for definitions). Representative docking poses from the top 6 obtained for all 18 protein – Hp/HS oligosaccharide complexes are shown here. Redundant poses are not presented for clarity. Native poses in each both are shown in green, while docked poses are in orange.

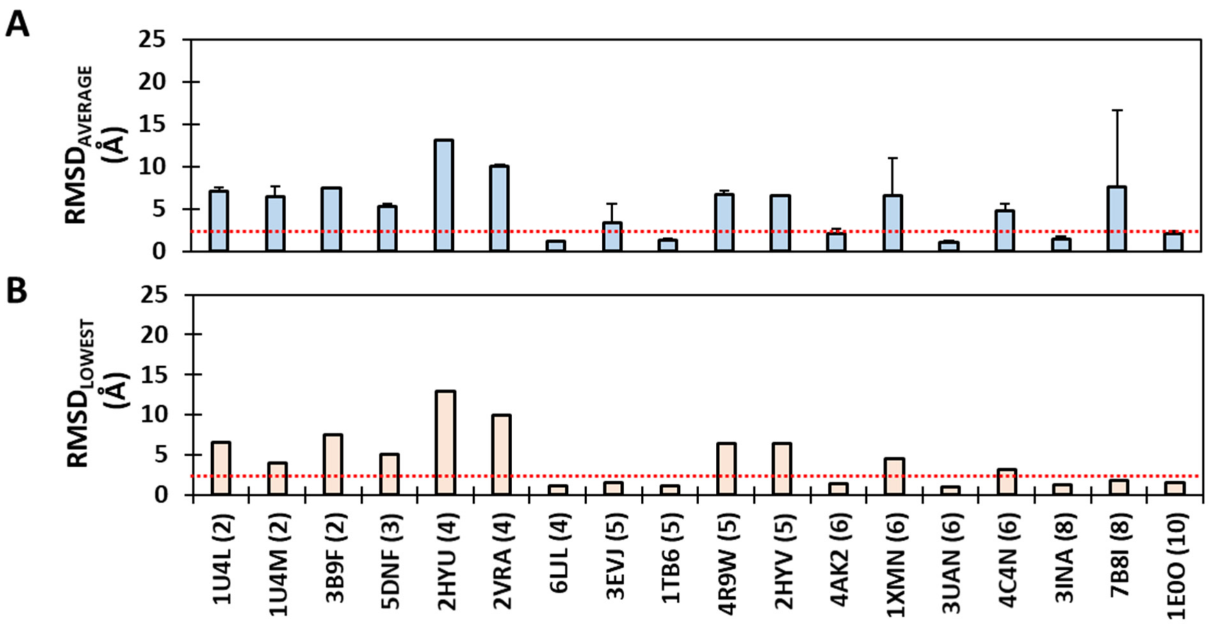

Figure S5. Recapitulation of the native pose using the rigid docking protocol using 300 GA runs. Each GA run was allowed 100,000 genetic operations. The top two poses from each replicate experiment were selected, compiled and used for analysis. The docking of each Hp/HS oligosaccharide onto its target protein was analyzed by calculating the $RMSD_{AVERAGE}$ and $RMSD_{LOWEST}$. Plots of $RMSD_{AVERAGE}$ (A) and $RMSD_{LOWEST}$ (B) as a function IDs of the co-complex structures reported in the PDB. X-axis labels represent the PDB code followed by chain length in brackets. Red dotted line indicates the 2.5 Å cutoff.

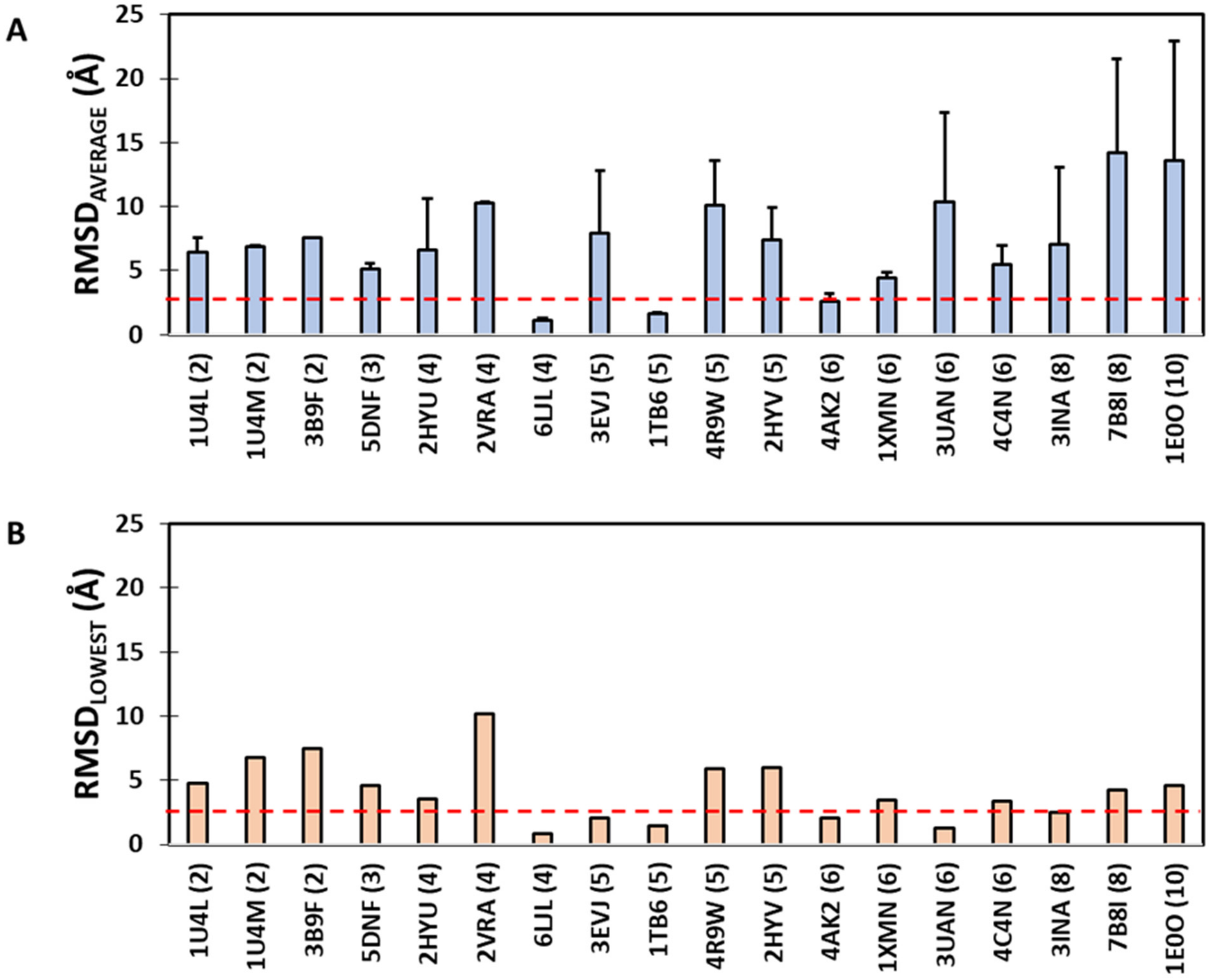

Figure S6. Recapitulation of the native pose using the flexible docking protocol using 300 GA runs. Each GA run was allowed 100,000 genetic operations. The top two poses from each replicate experiment were selected, compiled and used for analysis. The docking of each Hp/HS oligosaccharide onto its target protein was analyzed by calculating the RMSD$_{AVERAGE}$ and RMSD$_{LOWEST}$. Plots of RMSD$_{AVERAGE}$ (A) and RMSD$_{LOWEST}$ (B) as a function IDs of the co-complex structures reported in the PDB. X-axis labels represent the PDB code followed by chain length in brackets. Red dotted line indicates the 2.5 Å cutoff.

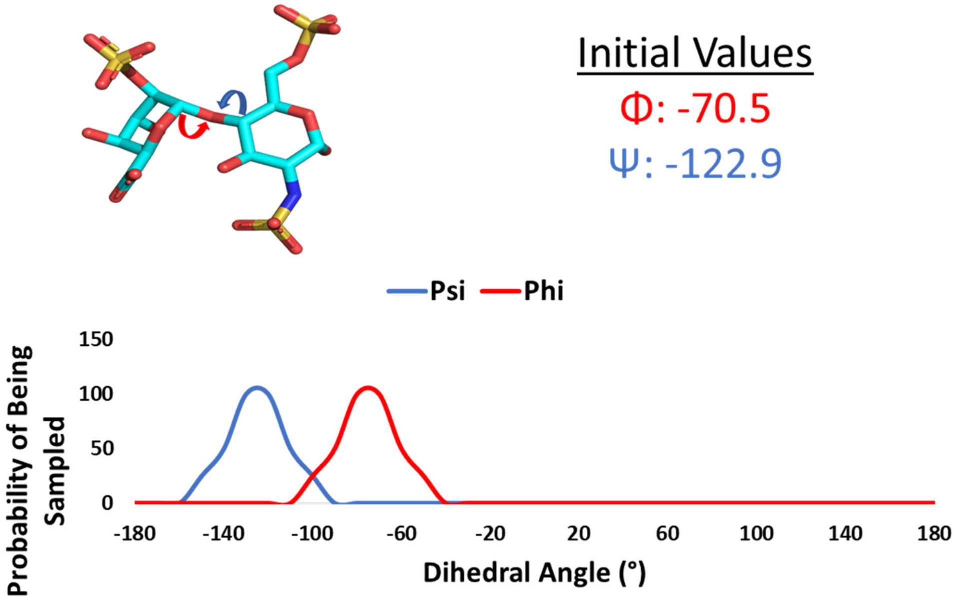

Example Torsional Histogram for Φ:

Phi
O.3(C.3(C.2)) | C.3 | O.3 | C.3(C.3(O.3(C.3(O.3(1H)))))

0	0	0	0	0	25	50	100	100	50	25	0	0	
0	0	0	0	0	0	0	0	0	0	0	0	0	0
0	0	0	0	0	0	0	0	0	0				

1) Torsion name
2) Four atoms specifying the torsion. To specify a uniqe atom, start with that atom first, then include atoms connected directly to the atom of interest by using parentheses.
3) A list of bins that represents the degree range starting from -180 to 180. The number in each bin is the probability that an angle in that bin will be sampled.

Figure S7. An example showing the torsional probability distribution function used in this work for semi-rigid docking protocol.

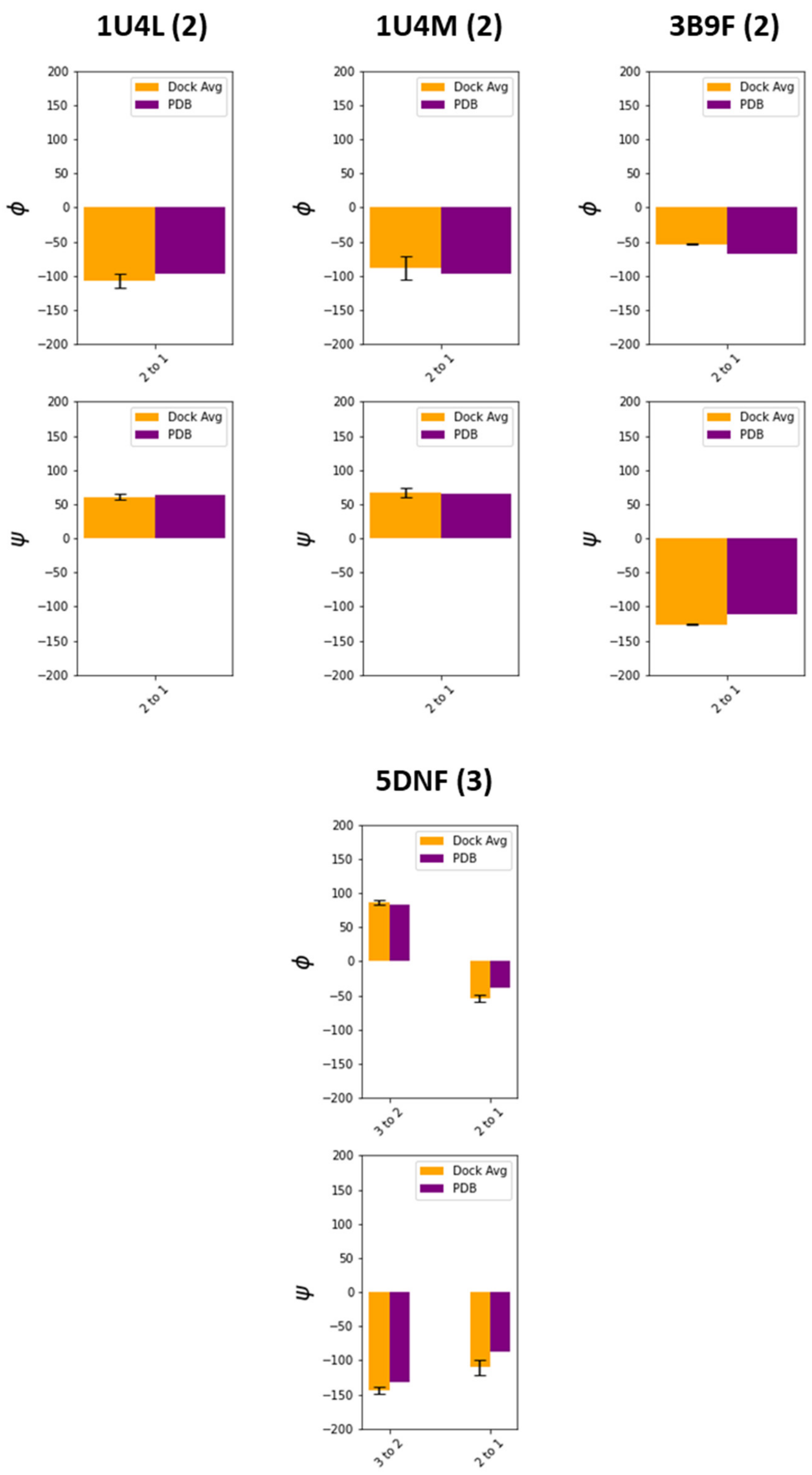

Figure S8. Comparison of the Φ/Ψ in the native pose with that observed following semi-rigid docking for two disaccharides and one trisaccharide of the 18 co-complexes studied in this work. Reducing end residue is labeled as 1 followed by residue labeling in increasing order. Glycosidic linkages are depicted as 1→2, 2→3, and so on.

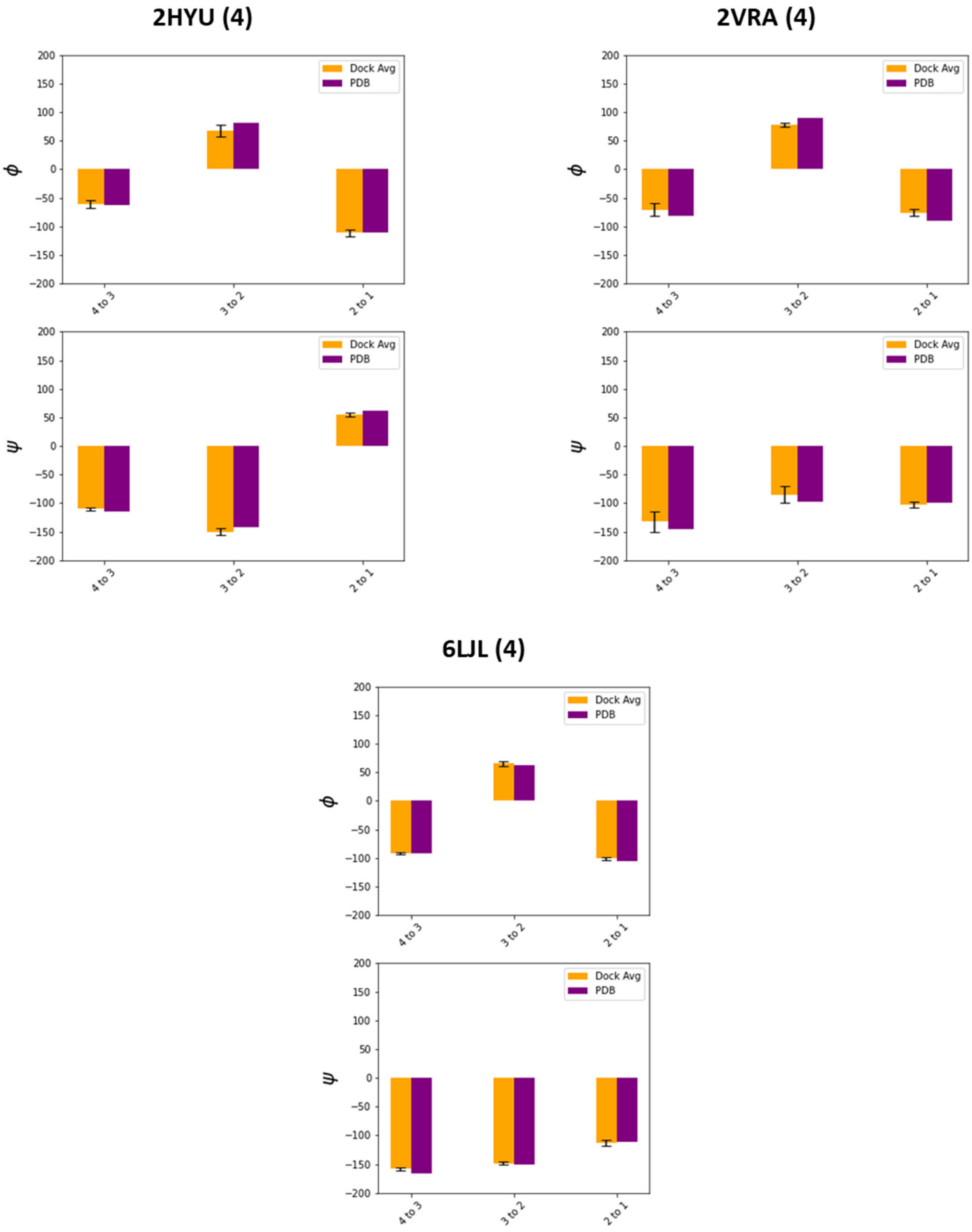

Figure S9. Comparison of the Φ/Ψ in the native pose with that observed following semi-rigid docking for three tetrasaccharides of the 18 co-complexes studied in this work. Reducing end residue is labeled as 1 followed by residue labeling in increasing order. Glycosidic linkages are depicted as 1→2, 2→3, and so on.

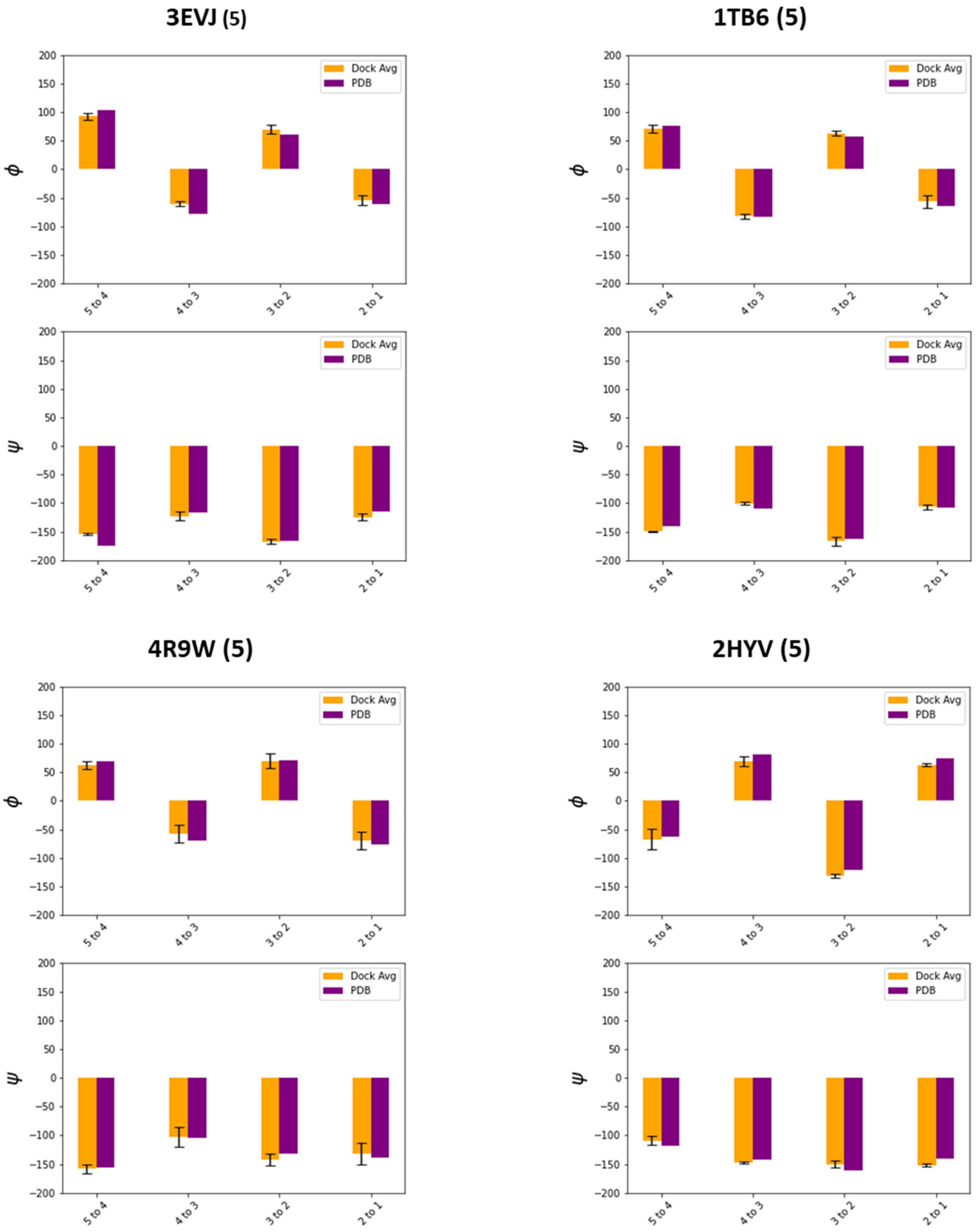

Figure S10. Comparison of the Φ/Ψ in the native pose with that observed following semi-rigid docking for four pentasaccharides of the 18 co-complexes studied in this work. Reducing end residue is labeled as 1 followed by residue labeling in increasing order. Glycosidic linkages are depicted as 1→2, 2→3, and so on.

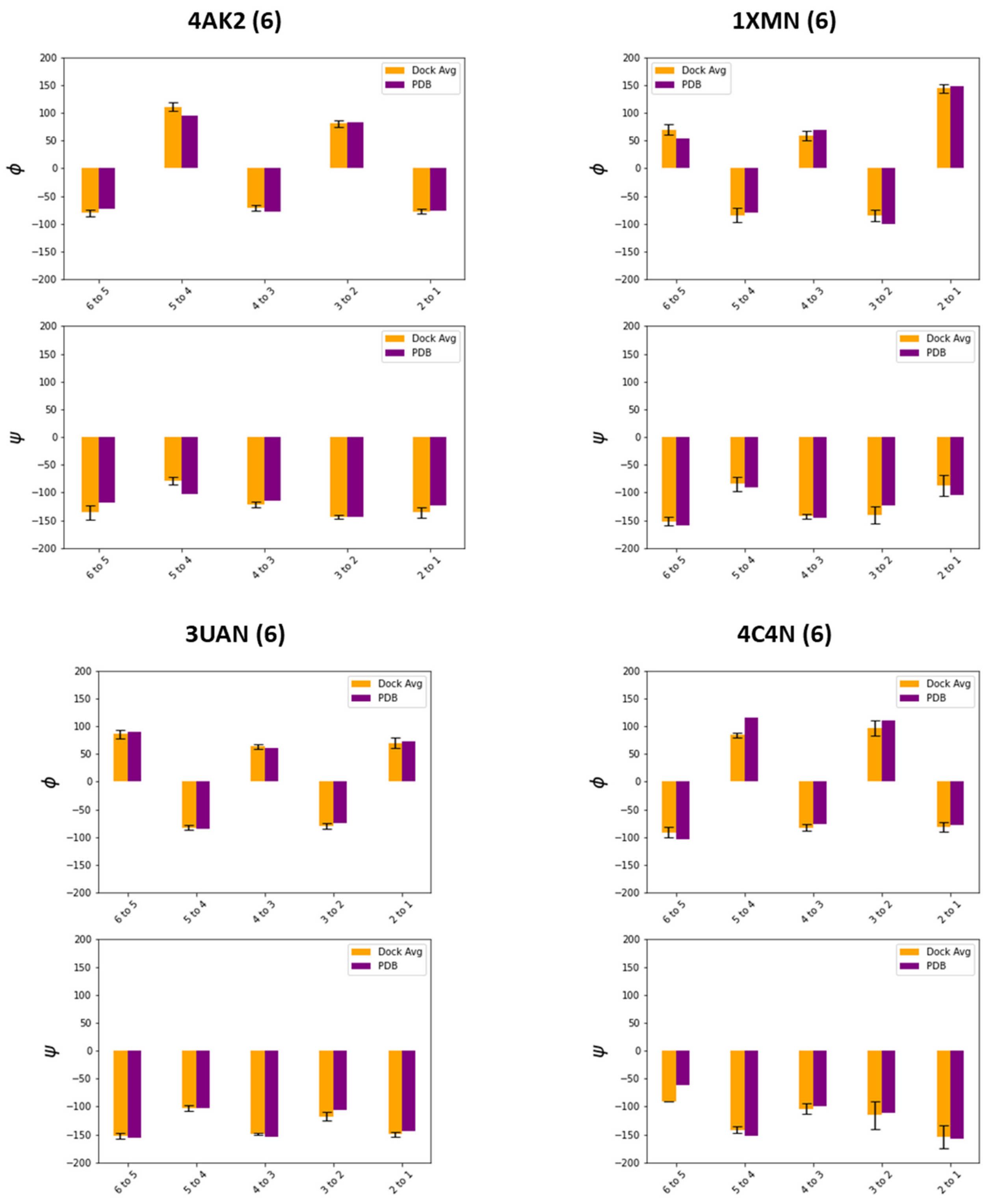

Figure S11. Comparison of the Φ/Ψ in the native pose with that observed following semi-rigid docking for four hexasaccharides of the 18 co-complexes studied in this work. Reducing end residue is labeled as 1 followed by residue labeling in increasing order. Glycosidic linkages are depicted as 1→2, 2→3, and so on.

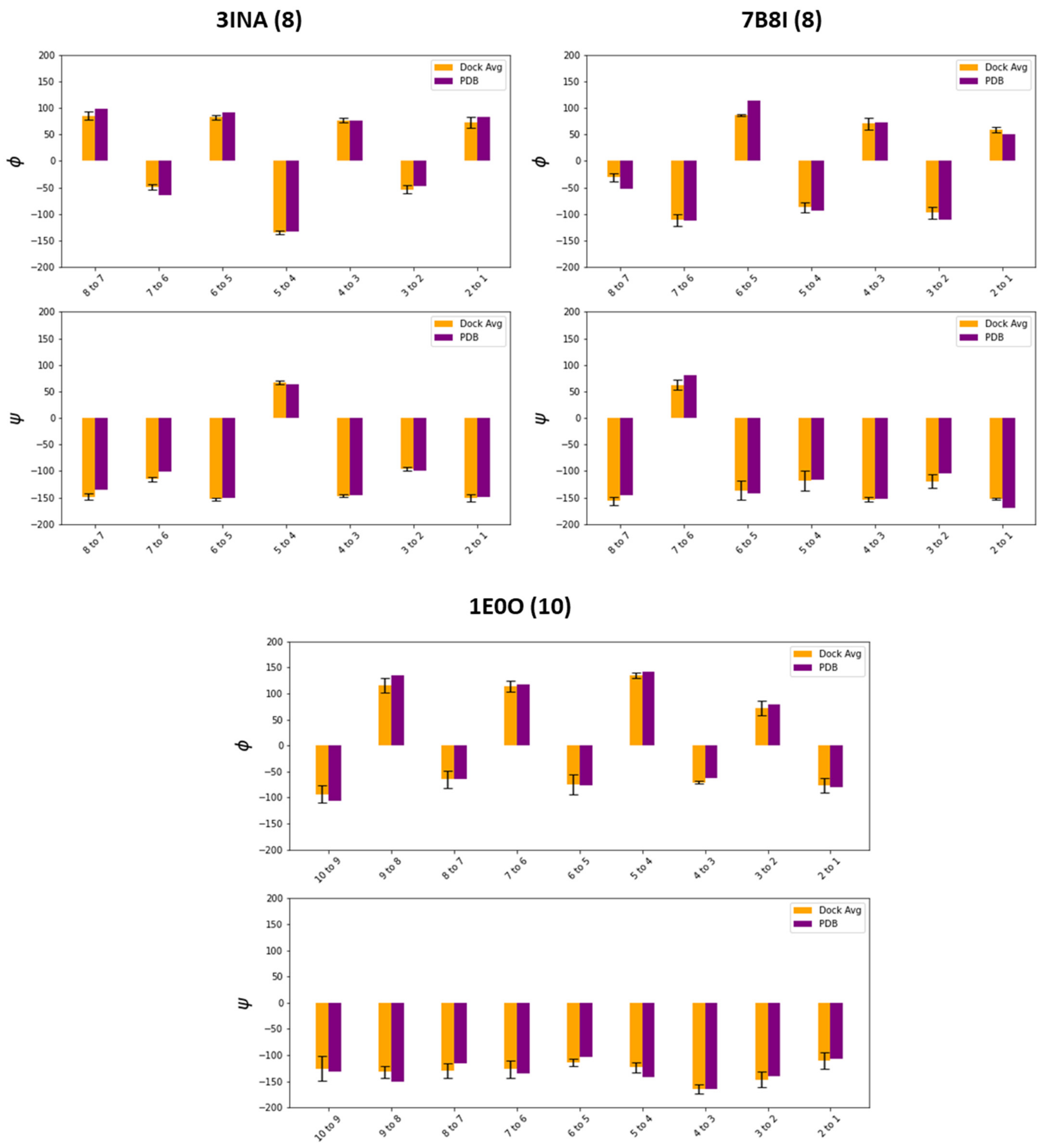

Figure S12. Comparison of the Φ/Ψ in the native pose with that observed following semi-rigid docking for two octasaccharides and one decasaccharide of the 18 co-complexes studied in this work. Reducing end residue is labeled as 1 followed by residue labeling in increasing order. Glycosidic linkages are depicted as 1→2, 2→3, and so on.

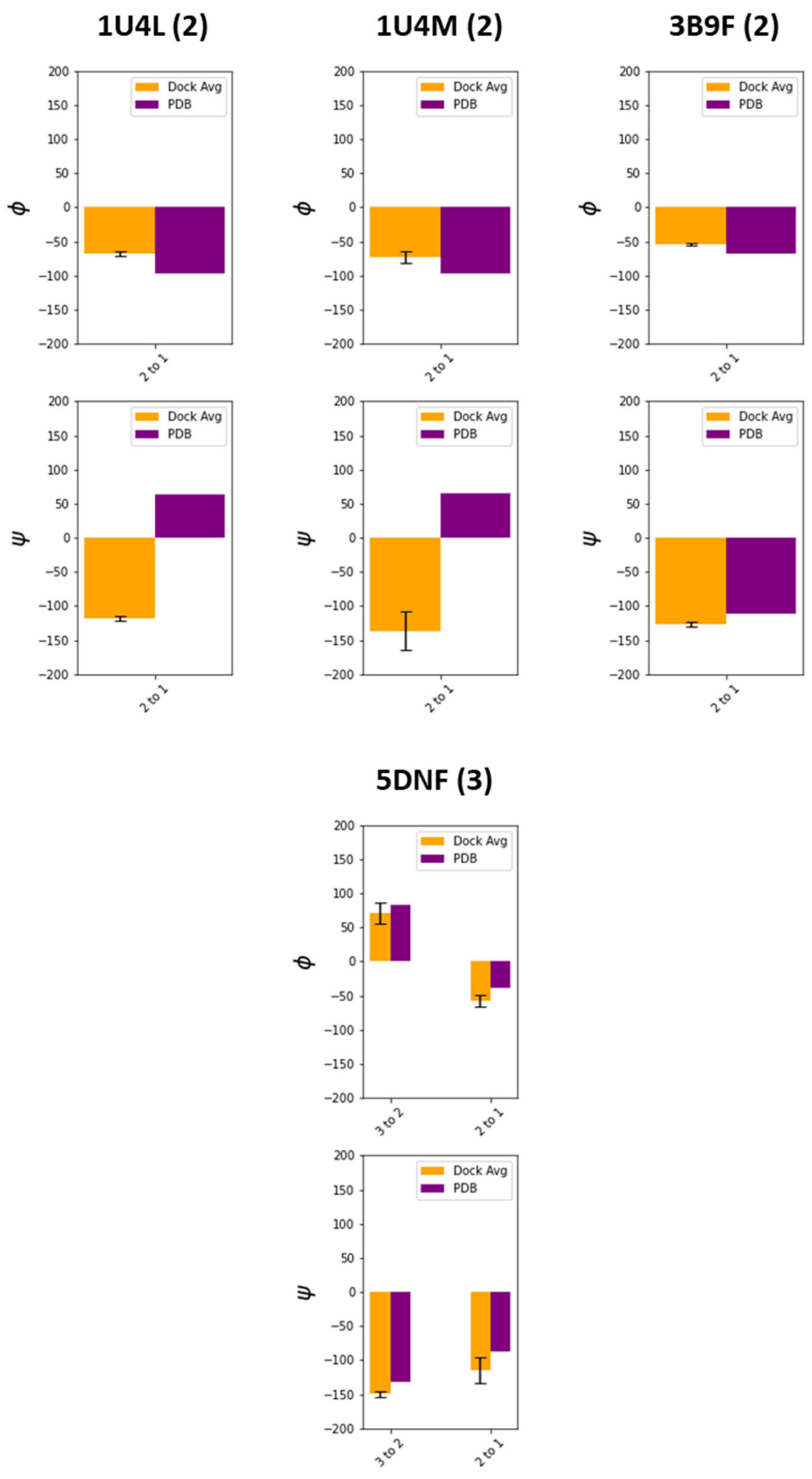

Figure S13. Comparison of the Φ/Ψ in the native pose with that observed following flexible docking for two disaccharides and one trisaccharide of the 18 co-complexes studied in this work. Reducing end residue is labeled as 1 followed by residue labeling in increasing order. Glycosidic linkages are depicted as 1→2, 2→3, and so on.

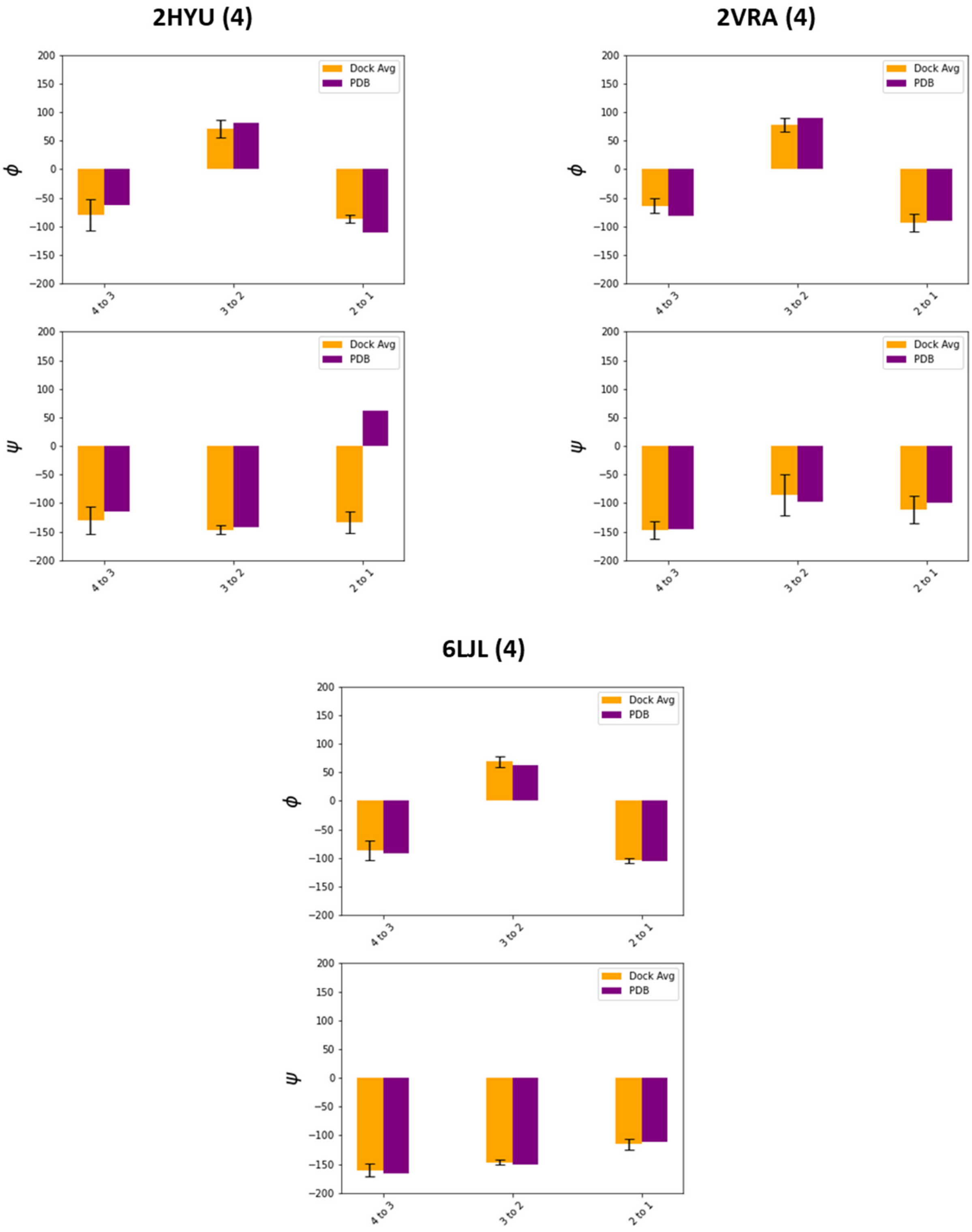

Figure S14. Comparison of the Φ/Ψ in the native pose with that observed following flexible docking for three tetrasaccharides of the 18 co-complexes studied in this work. Reducing end residue is labeled as 1 followed by residue labeling in increasing order. Glycosidic linkages are depicted as 1→2, 2→3, and so on.

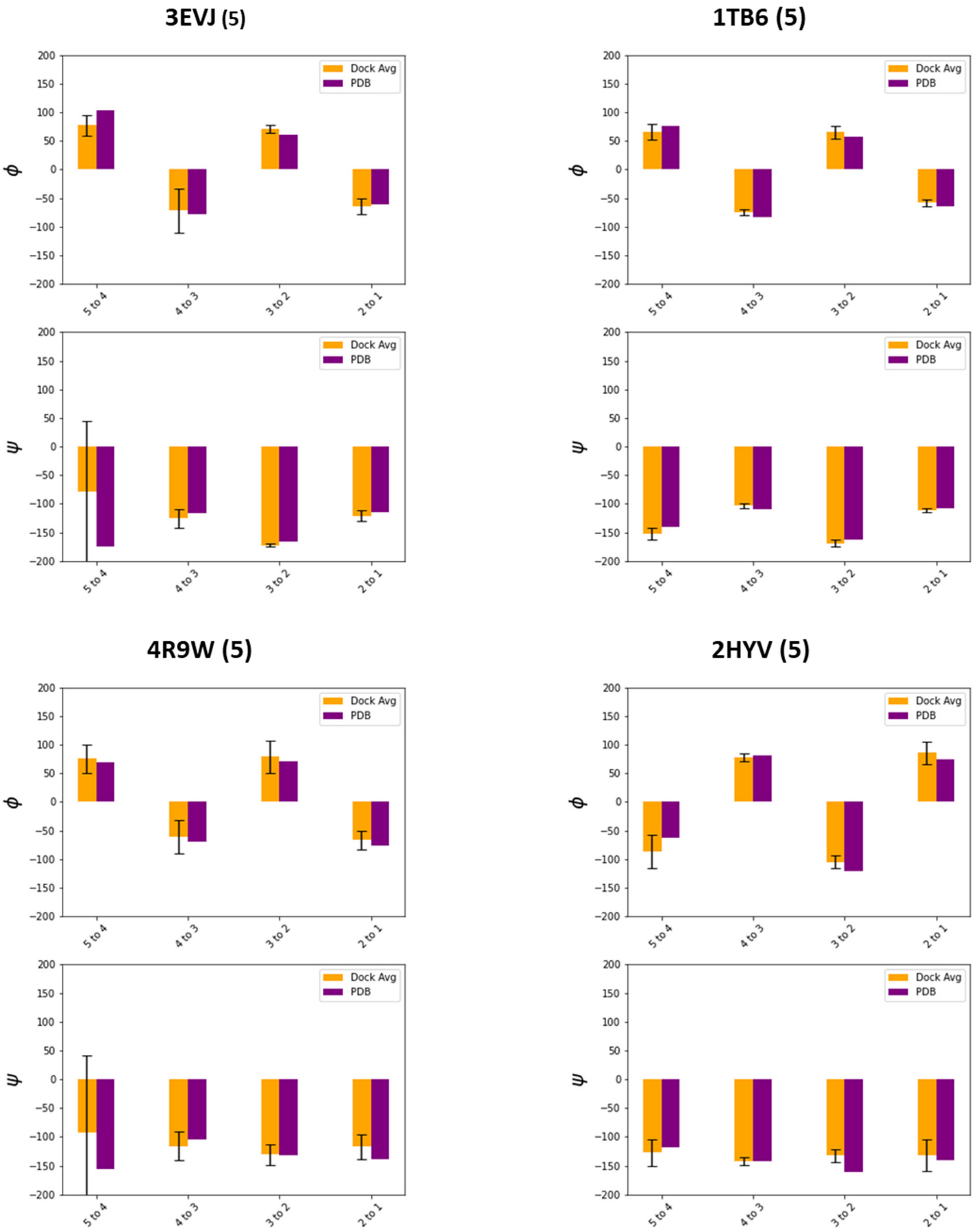

Figure S15. Comparison of the Φ/Ψ in the native pose with that observed following flexible docking for four pentasaccharides of the 18 co-complexes studied in this work. Reducing end residue is labeled as 1 followed by residue labeling in increasing order. Glycosidic linkages are depicted as 1→2, 2→3, and so on.

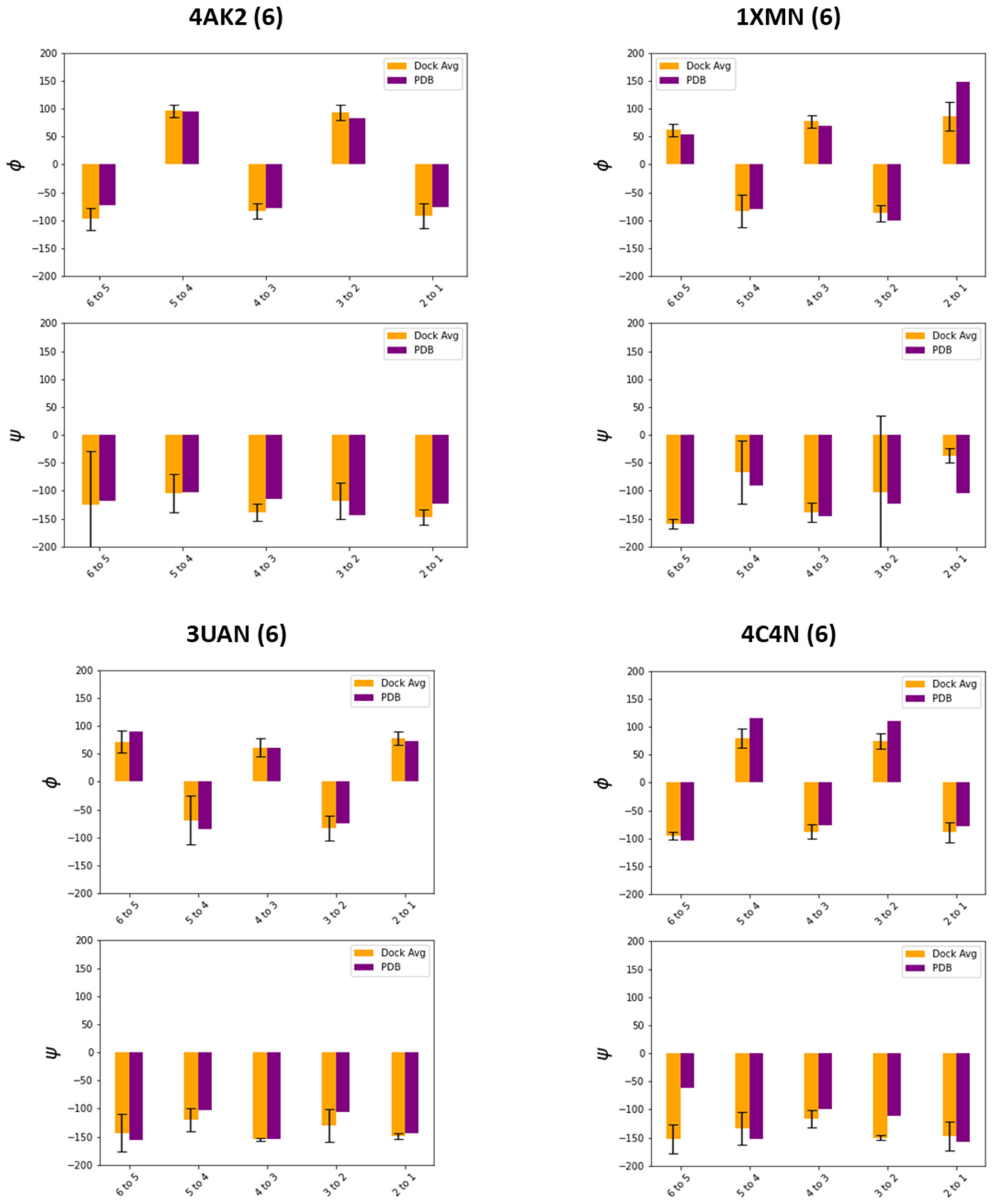

Figure S16. Comparison of the Φ/Ψ in the native pose with that observed following flexible docking for four hexasaccharides of the 18 co-complexes studied in this work. Reducing end residue is labeled as 1 followed by residue labeling in increasing order. Glycosidic linkages are depicted as 1→2, 2→3, and so on.

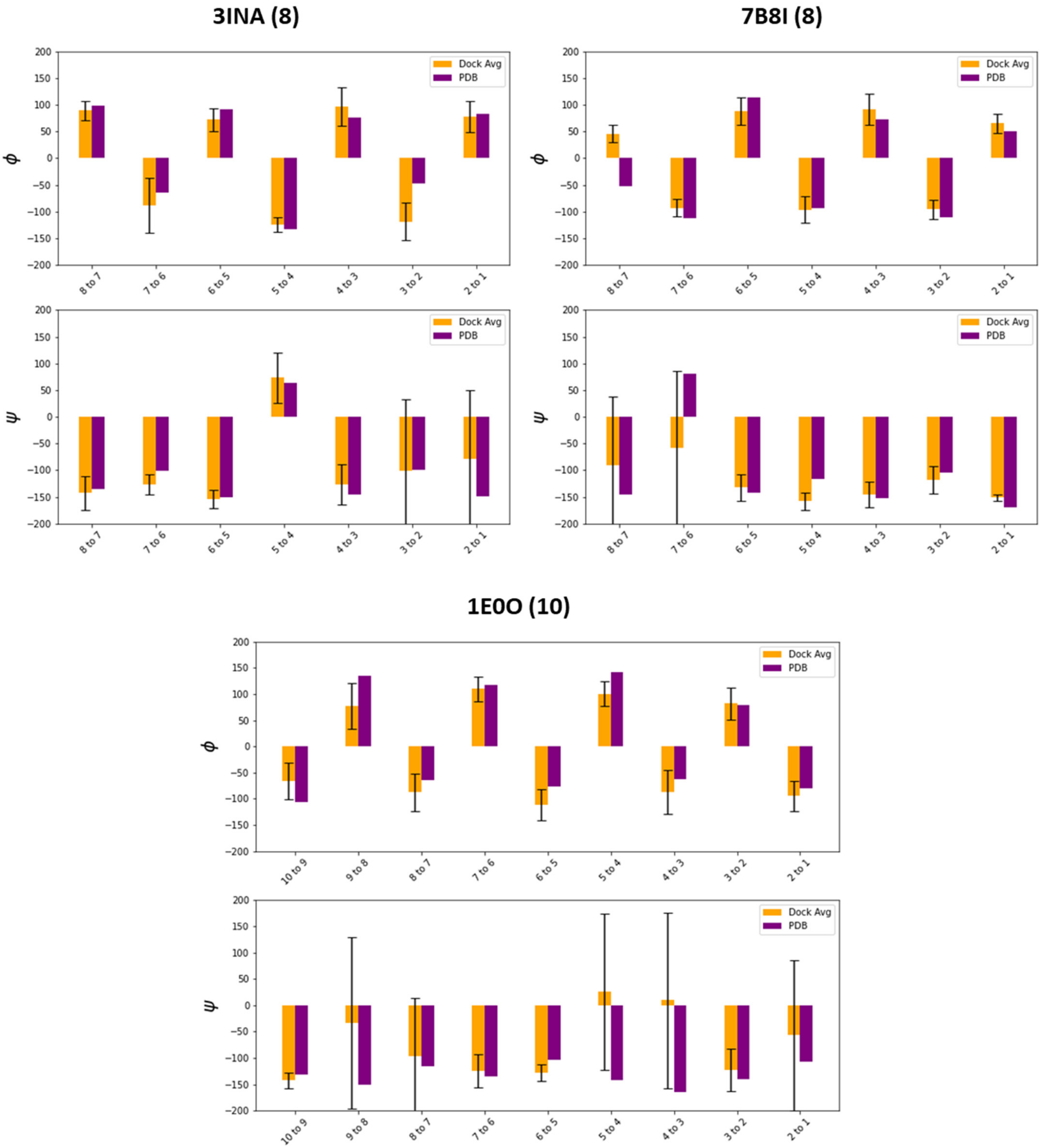

Figure S17. Comparison of the Φ/Ψ in the native pose with that observed following flexible docking for two octasaccharides and one decasaccharide of the 18 co-complexes studied in this work. Reducing end residue is labeled as 1 followed by residue labeling in increasing order. Glycosidic linkages are depicted as 1→2, 2→3, and so on.

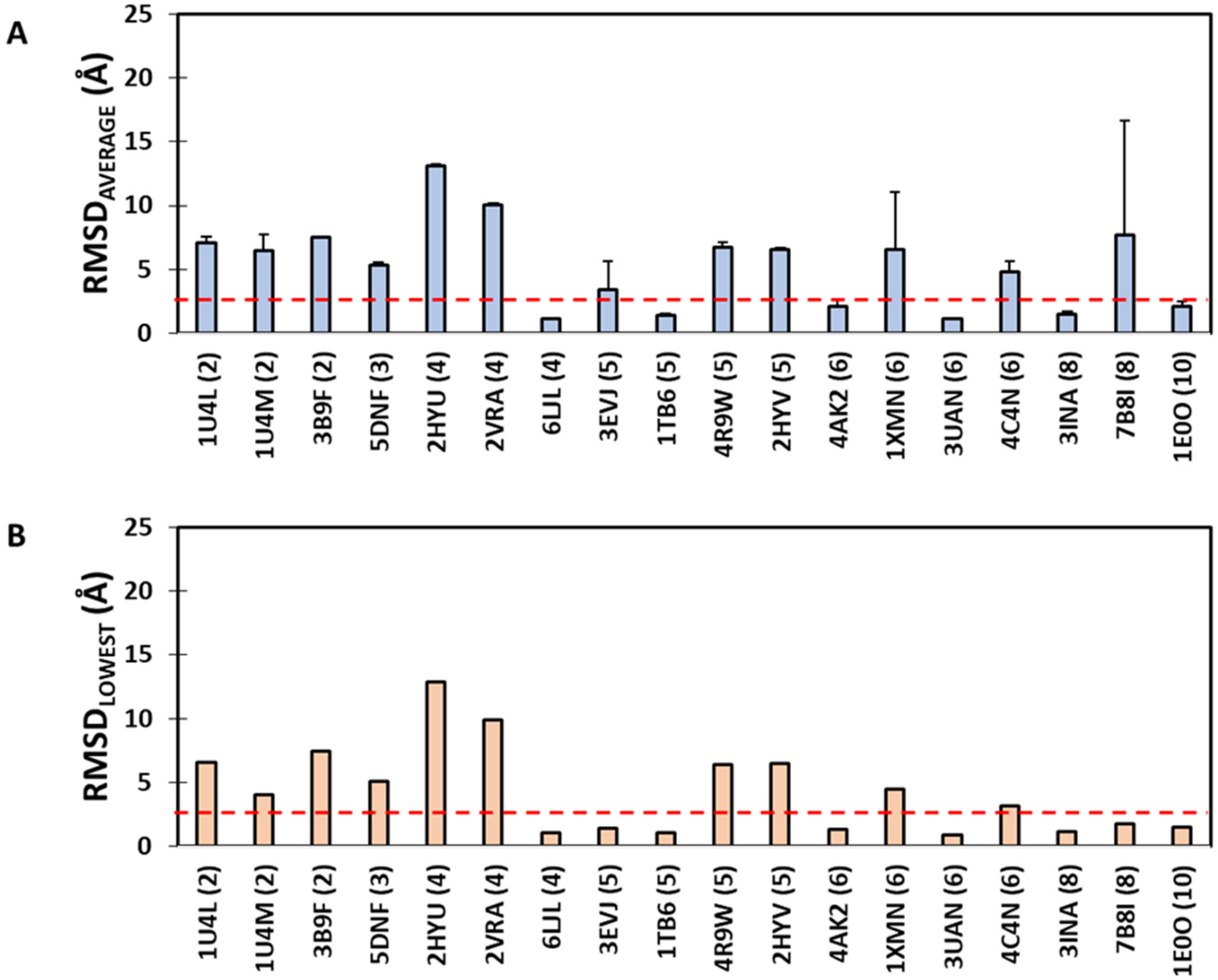

Figure S18. Recapitulation of the native pose using the semi-rigid docking protocol using 300 GA runs. Each GA run was allowed 100,000 genetic operations. The top two poses from each replicate experiment were selected, compiled and used for analysis. The docking of each Hp/HS oligosaccharide onto its target protein was analyzed by calculating the $RMSD_{AVERAGE}$ and $RMSD_{LOWEST}$. Plots of $RMSD_{AVERAGE}$ (A) and $RMSD_{LOWEST}$ (B) as a function IDs of the co-complex structures reported in the PDB. X-axis labels represent the PDB code followed by chain length in brackets. Red dotted line indicates the 2.5 Å cutoff.

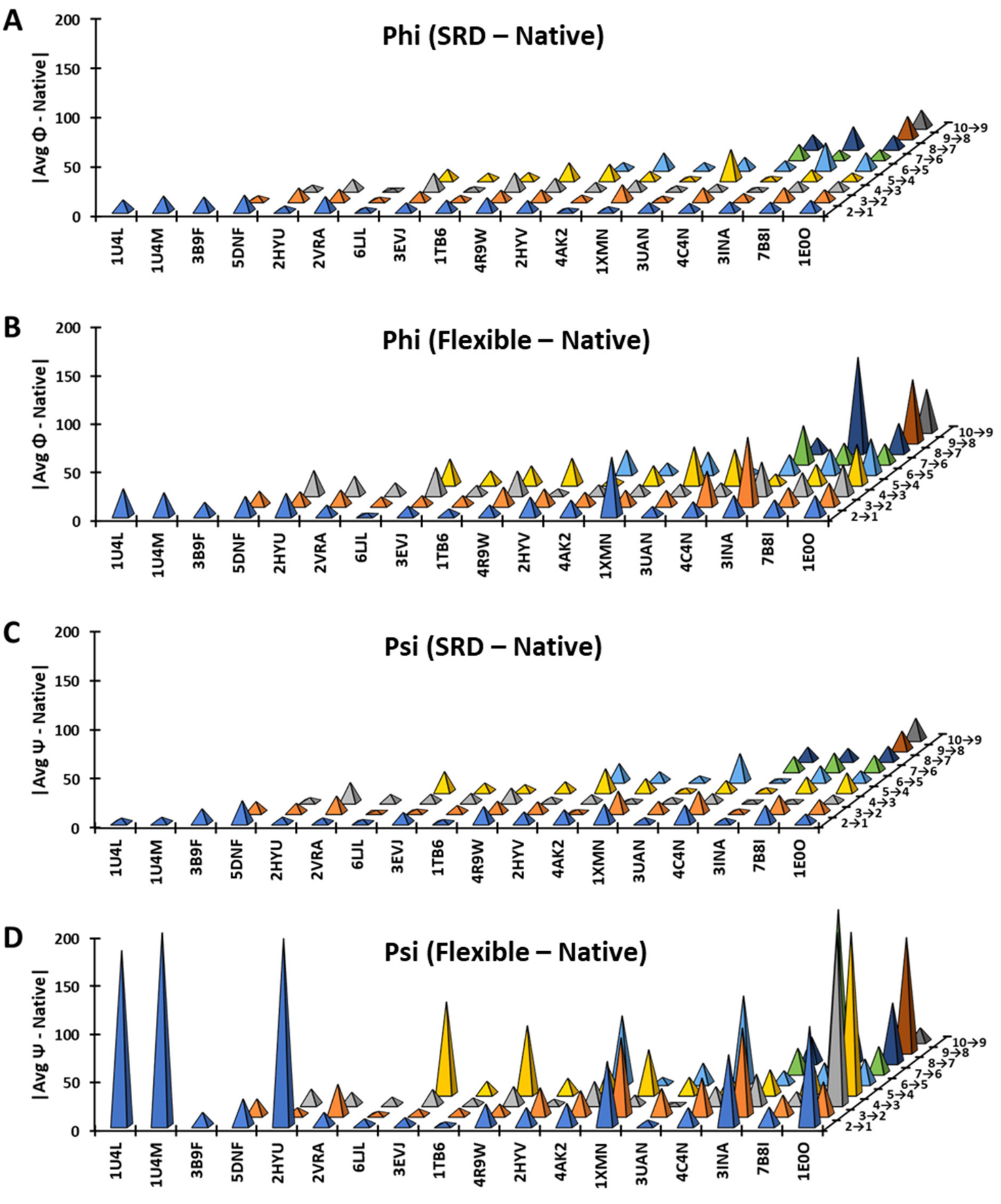

Figure S19. Comparison of changes in Φ/Ψ from their values in the native state following for semi-rigid and flexible dockings. Note: Change in Φ/Ψ can be either positive or negative, yet only the degree of change is depicted here. A) Change in Φ following SRD. B) Change in Φ following flexible docking. C) Change in Ψ following SRD. D) Change in Ψ following flexible docking.

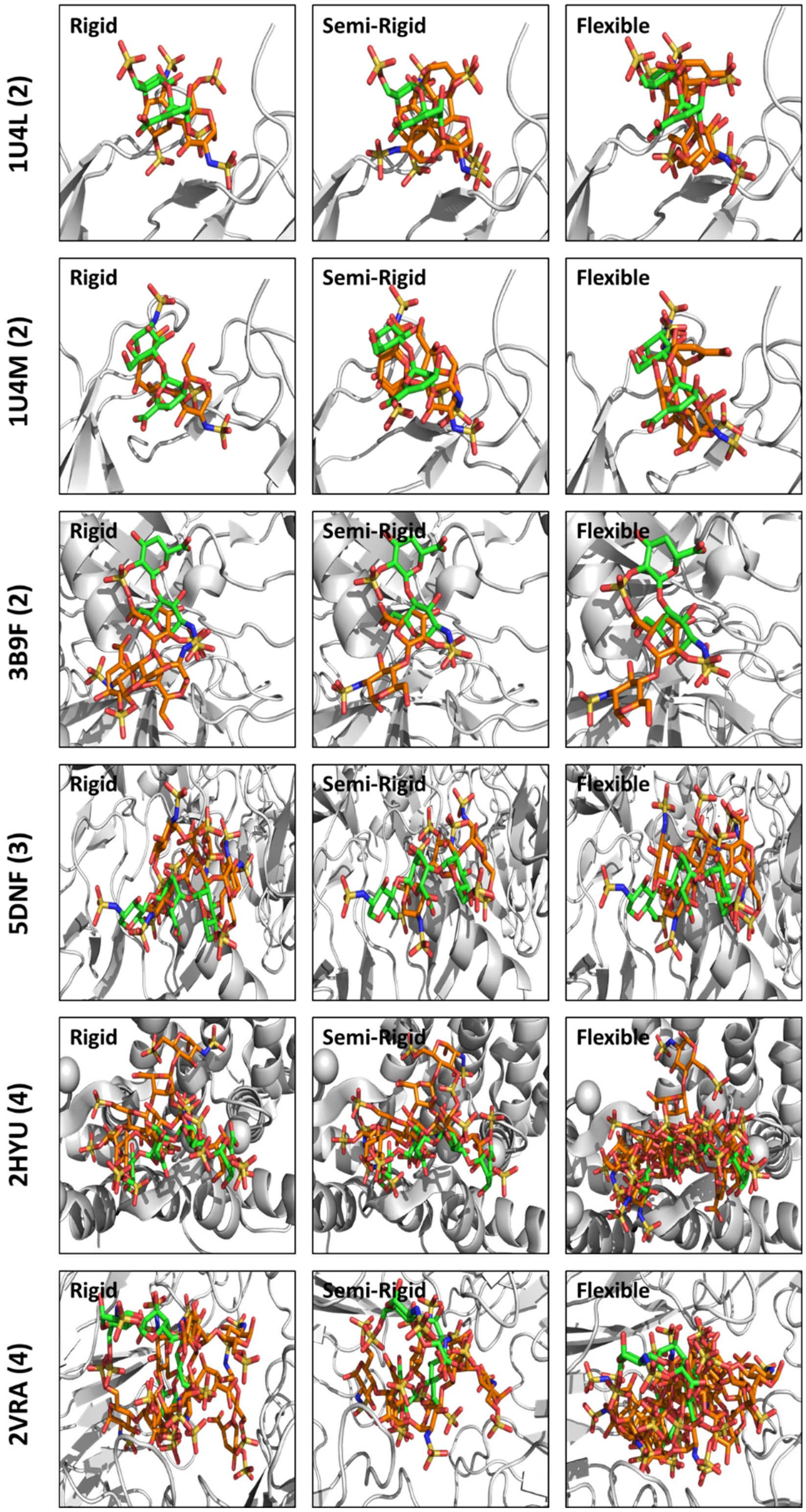

Figure S20. Comparison of docked poses using rigid, semi-rigid, and flexible docking protocols to the native pose observed in the co-crystal structures of three di-, one tri- and two tetra- saccharides. Redundant poses have been excluded for clarity. Proteins are shown in cartoon representation in grey, the native pose in green, and docked poses in orange. Models were generated in PyMOL.

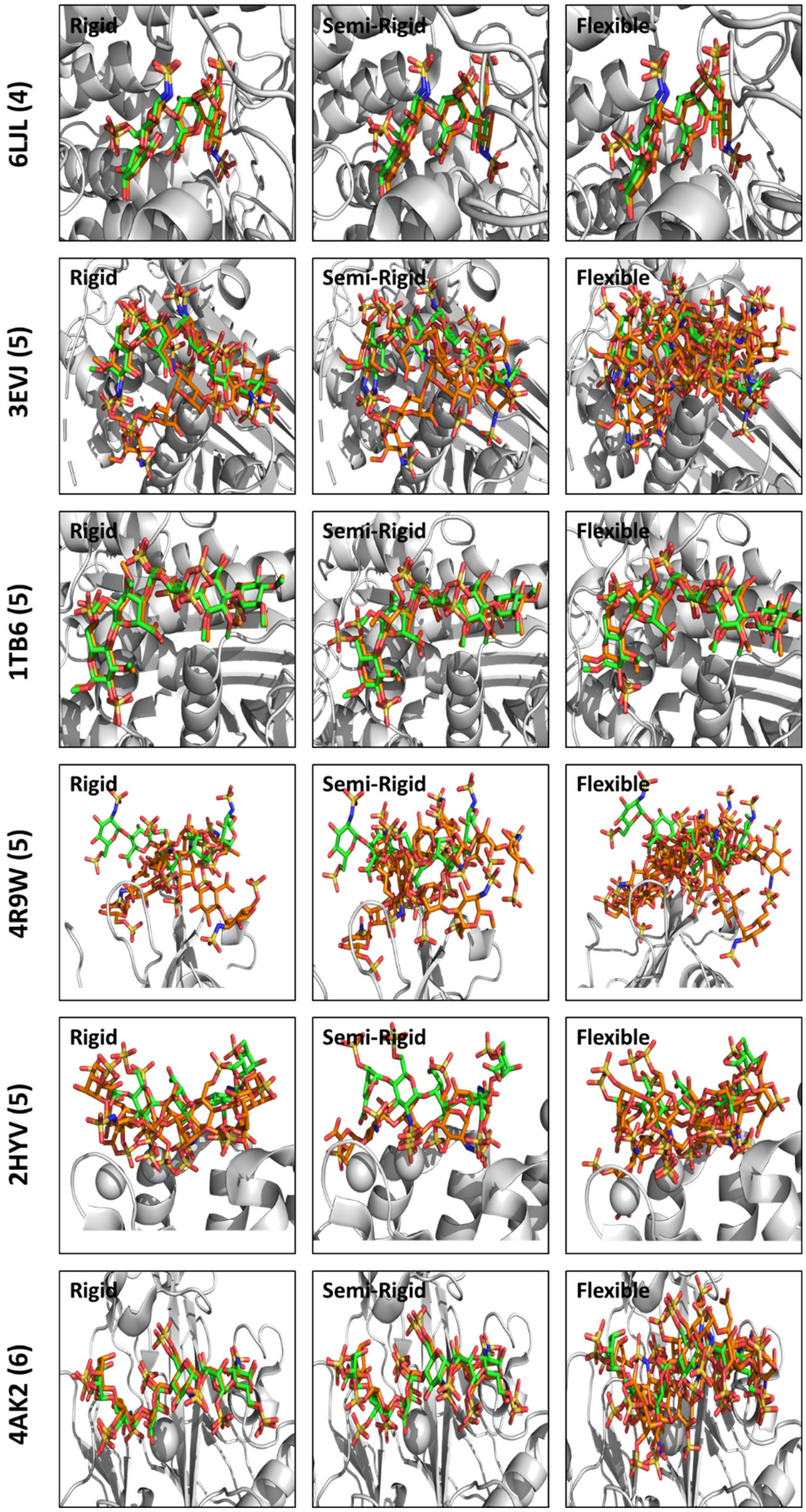

Figure S21. Comparison of docked poses using rigid, semi-rigid, and flexible docking protocols to the native pose observed in the co-crystal structures of one tetra-, four penta- and one hexa- saccharides. Redundant poses have been excluded for clarity. Proteins are shown in cartoon representation in grey, the native pose in green, and docked poses in orange. Models were generated in PyMOL.

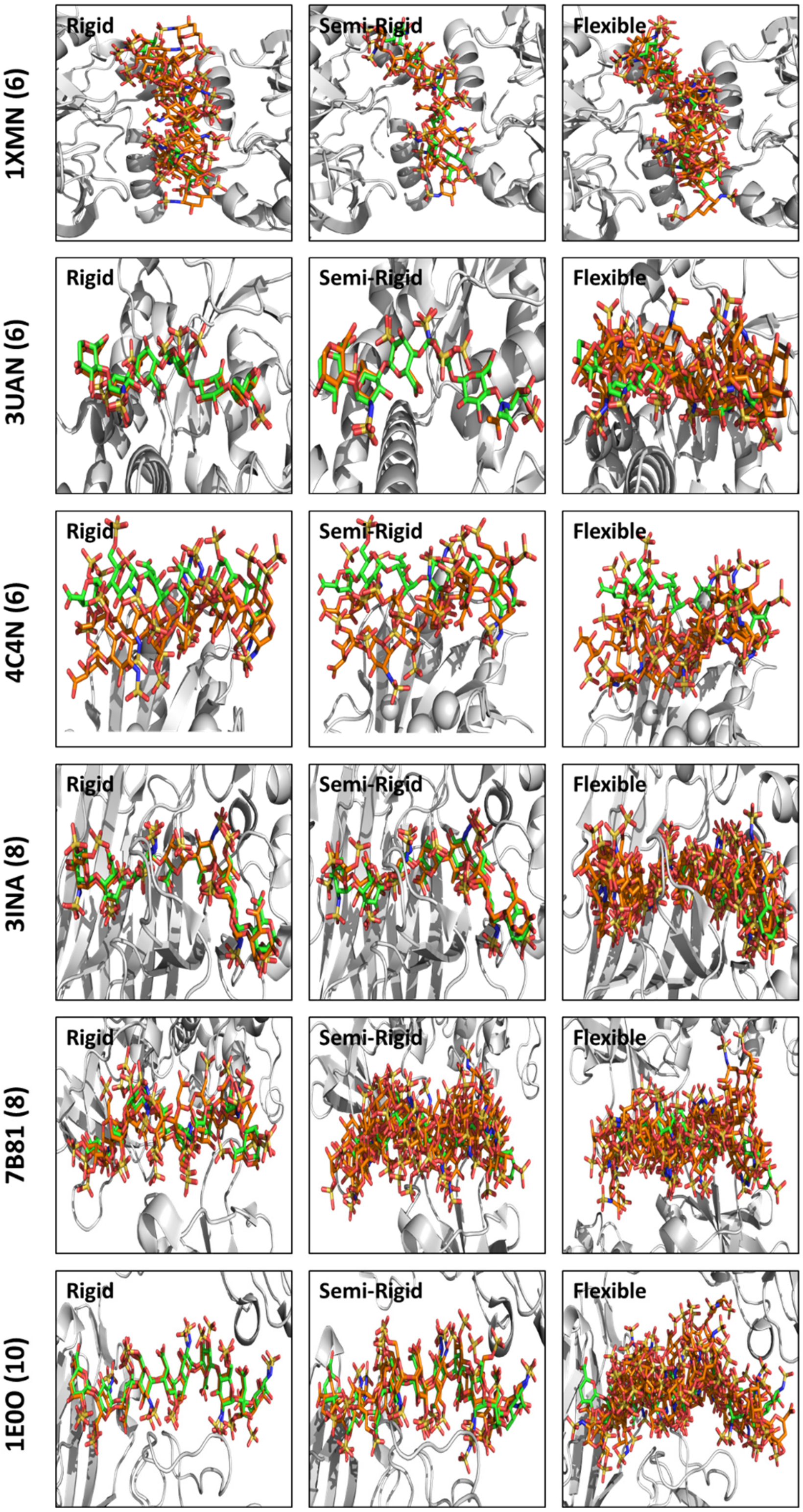

Figure S22. Comparison of docked poses using rigid, semi-rigid, and flexible docking protocols to the native pose observed in the co-crystal structures of three hexa-, two octa- and one deca- saccharides. Redundant poses have been excluded for clarity. Proteins are shown in cartoon representation in grey, the native pose in green, and docked poses in orange. Models were generated in PyMOL.
